# Global Analysis of Small RNA Dynamics during Seed Development of *Picea glauca* and *Arabidopsis thaliana* Populations Reveals Insights on their Evolutionary Trajectories

**DOI:** 10.3389/fpls.2017.01719

**Published:** 2017-10-04

**Authors:** Yang Liu, Yousry A. El-Kassaby

**Affiliations:** Department of Forest and Conservation Sciences, University of British Columbia, Vancouver, BC, Canada

**Keywords:** adaptive strategy, *Arabidopsis thaliana*, microRNA, organismal complexity, phenotypic variation, *Picea glauca*, seed ontogeny, small RNA evolution

## Abstract

While DNA methylation carries genetic signals and is instrumental in the evolution of organismal complexity, small RNAs (sRNAs), ~18–24 ribonucleotide (nt) sequences, are crucial mediators of methylation as well as gene silencing. However, scant study deals with sRNA evolution via featuring their expression dynamics coupled with species of different evolutionary time. Here we report an atlas of sRNAs and microRNAs (miRNAs, single-stranded sRNAs) produced over time at seed-set of two major spermatophytes represented by populations of *Picea glauca* and *Arabidopsis thaliana* with different seed-set duration. We applied diverse profiling methods to examine sRNA and miRNA features, including size distribution, sequence conservation and reproduction-specific regulation, as well as to predict their putative targets. The top 27 most abundant miRNAs were highly overlapped between the two species (e.g., miR166,−319 and−396), but in *P. glauca*, they were less abundant and significantly less correlated with seed-set phases. The most abundant sRNAs in libraries were deeply conserved miRNAs in the plant kingdom for *Arabidopsis* but long sRNAs (24-nt) for *P. glauca*. We also found significant difference in normalized expression between populations for population-specific sRNAs but not for lineage-specific ones. Moreover, lineage-specific sRNAs were enriched in the 21-nt size class. This pattern is consistent in both species and alludes to a specific type of sRNAs (e.g., miRNA, tasiRNA) being selected for. In addition, we deemed 24 and 9 sRNAs in *P. glauca* and *Arabidopsis*, respectively, as sRNA candidates targeting known adaptive genes. Temperature had significant influence on selected gene and miRNA expression at seed development in both species. This study increases our integrated understanding of sRNA evolution and its potential link to genomic architecture (e.g., sRNA derivation from genome and sRNA-mediated genomic events) and organismal complexity (e.g., association between different sRNA expression and their functionality).

## Introduction

Epigenetic mechanisms exert functional roles in the upper dimension of gene regulatory networks (GRNs) and are more important to the transcription machinery than have hitherto been thought. Because changes alone in epigenetics can affect complex traits across generations in the absence of genetic variation (Cubas et al., 1999; Johannes et al., 2009; Richards et al., 2012), where salient examples of the direct impact of epimutations (e.g., epQTLs, modified polymorphisms in nucleotides) on phenotypic variation are mainly instantiated in the profiling of epialleles (i.e., single-locus DNA methylation variants). While non-coding small RNA (sRNA) molecules are paramount to genomic DNA methylation via sequence-specific recognition to recruit regulatory proteins in the RNA-directed DNA methylation (RdDM) pathway (Lewsey et al., 2016), there are, supposedly, parent-offspring transmission patterns of methylation marks and sRNAs [e.g., small interfering RNAs (siRNAs)], as put forth by Diez et al. (2014). Moreover, the expression level of protein-encoding genes (PEGs) correlates with the density of their nearby methylated transposable elements (TEs) in accessible euchromatic regions, reviewed by Diez et al. (2014) and Sigman and Slotkin (2016). sRNAs particularly in the 24 nucleotide (nt) size class are crucial regulators of TEs (Almeida and Allshire, 2005; Nosaka et al., 2012) and have natural origins from TE segments (Piriyapongsa and Jordan, 2008; Sun et al., 2012). It is therefore highly conceivable that there are tight associations between sRNAs (type and abundance) and gene expression regulation in shaping regulatory diversity and robustness.

Plant sRNAs are generated from stem-loop regions of longer primary transcripts (or fold-back structures from single- or double-stranded RNA precursors) by Dicer-like enzymes (DCLs) and chiefly comprise microRNAs [miRNAs; prevalence of 21- or 22-nt long in suppressing target mRNAs], heterochromatic siRNAs [hc-siRNAs; 24-nt mediators in silencing DNA methylation and histone modifications], and *trans*-acting siRNAs [tasiRNAs or phasiRNAs; 22 (or 21)-nt with a phased configuration, playing similar roles as miRNAs or other uncharacterized functions], reviewed by Wendel et al. (2016). As sRNAs form duplexes when derived or pairing with another sRNA or binding to mRNA to direct cleavage and degradation, the capability to pair with other genomic sequence(s) is their fundamentally common feature. At the levels of transcription (indirect and low) and post-transcription (major), miRNAs are well-established players in various developmental programs (Dugas and Bartel, 2004; Sparks et al., 2013) and plasticity (Rubio-Somoza and Weigel, 2011). In response to environmental cues, GRNs can be overridden via instigating the biogenesis of different miRNAs. In major land plant lineages from the backbone phylogenetic tree, acquisition of different miRNA families has been summarized using parsimony approaches and only a handful of distinct families of deeply conserved miRNAs are evolutionarily ancient and stable (Zhang et al., 2006; Axtell et al., 2007; Axtell and Bowman, 2008). Large diverse sets of lineage-specific miRNAs often exceed the conserved ones (Lindow and Krogh, 2005; Cuperus et al., 2011). Presumably, myriad *MIRNA*s (miRNA genes) are expanded but unrevealed (Fattash et al., 2007; Nozawa et al., 2012), or young *MIRNA*s are spawned, weakly expressed and eventually frequently lost (Fahlgren et al., 2007). This indicates that lineage-specific miRNAs have undergone rapid turnover in evolution (e.g., 1.2–3.3 genes per Myr in Arabidopsis, Fahlgren et al., 2010) and *MIRNA*s evolve adaptively, possibly driven by positive selection. From the phylogenetic perspective, distribution of miRNA families seems to be approximately proportional to the antiquity of the evolutionary lineages (Taylor et al., 2014). Novel miRNAs may adjust the variance of expression levels of their target genes to maintain the stability of transcription networks and after diversifying and purifying selection, they reset mean gene expression to improve fitness of specific phenotypes (Wu et al., 2009). Co-option of ancient and young miRNA families to conduct new functions is important in the evolution of new phenotypes (Taylor et al., 2014) or phenotypic differentiation. In addition, duplication of *MIRNA*s, especially those from whole-genome duplication (WGD), may be also conducive to miRNA diversity and their regulatory complexity (Maher et al., 2006; Vanneste et al., 2014), indicative of coevolution of *MIRNA*s, miRNAs and their targets in the context of WGD events (or genome evolution).

In this study, we chose populations (ecotypes) within two spermatophytes with contrasting seed-set time span (i.e., *Picea glauca* and *Arabidopsis thaliana*), which implies different seed developmental modes possibly contributing to acclimation and adaptive differentiation. Conifers (Pinophyta or Coniferales) are masters of adaptation due to their long endurance over periods of climatic sways (Jaramillo-Correa et al., 2004; Anderson et al., 2006; Tollefsrud et al., 2008) and wide geographic distribution (Wang and Ran, 2014). Their long generation time confines the ability of populations to respond to environmental stresses through genetic mechanisms (Bräutigam et al., 2013), while epigenetics is more labile, malleable and potentially reversible, and thus more closely aligned with the environmental exposure to create new combinations of variants (Lira-Medeiros et al., 2010; Schulz et al., 2014). Extant conifers (Pennsylvanian, 318-299 Myr ago), bearing enormous genome size (e.g., 200 × Arabidopsis), are evolutionarily twice as old as angiosperms (early Cretaceous, 146 Myr ago) (Schneider et al., 2004) but with, on average, seven times lower in rates of molecular evolution (De La Torre et al., 2017), and have undergone few WGDs or polypoidization (Leitch and Leitch, 2012). The contrasting differences in genome architecture suggest disparate evolutionary mechanisms of genome and epigenetic control between conifers and Arabidopsis (angiosperms). Conifers have high levels of methylation in PEGs, at ~40% in vegetative tissues and over 75% in megagametophytes in *Pinus taeda* (Takuno et al., 2016) and are found to yield 24-nt sRNAs only at reproductive tissues (Nystedt et al., 2013), supportive to high levels of epigenetic modifications at conifer seed set. In *Arabidopsis thaliana*, specific heritable methylation patterns account for 60–90% of the heritability of two studied complex traits, flowering time and primary root length (Cortijo et al., 2014). Moreover, lines of evidence in *Picea abies* and *P. taeda* have shown that environmental conditions at seed set can substantially affect progeny performance (Johnsen et al., 2005; Kvaalen and Johnsen, 2008) and this process is mediated by miRNAs (Oh et al., 2008; Yakovlev et al., 2010). Recently, it has been reported that environmental cues (e.g., temperature, CO_2_) alter the expression of miRNAs (e.g., miR156, −157, −160, −164, and −172) to regulate *Arabidopsis* development and growth (May et al., 2013). Together, sRNA dynamics encrypted at seed set may shed light on the plasticity potential in adaptation to habitat heterogeneity and plant life histories.

Local adaptation enables plants to obtain a high fitness and developmental transitions should be properly timed to coincide phases in plant growth with their favorable seasons. The seed is therefore a key evolutionary adaptation of seed plants that facilitates dispersal and reinitiates development only at suitable environmental conditions. Seeds have evolved the ability to start their life cycle at the right time via timing their germination. This timing in plant life history is controlled by seed dormancy (i.e., innate constraint on seed germination under conditions that would otherwise promote germination in non-dormant seeds), which is the target phenotype in this study. Plants use dormancy in seeds to move through time and space, which finally maximizes their fitness. Seed dormancy is under strong evolutionary selection, because improper timing of germination may lead to outright extinction (Huang et al., 2010). As post-zygotic quiescence may be the starting point of the evolution of seed dormancy (Mapes et al., 1989), dormancy modulation is assumed to be the consequence of finely tuned programs at seed set. We therefore hypothesize that selected populations can represent different modes of reproductive development that are regulated by sRNAs and exert cascading effects on ensuing phenotypes, including seed dormancy. We focus on miRNA-mRNA nodes responsible for seed dormancy formation (Liu and El-Kassaby, 2017a) and three key conserved genes [i.e., *ABA INSENSITIVE 3* (*ABI3*), *AUXIN RESPONSE FACTOR 10/16* (*ARF10/16*), and *DELAY OF GERMINATION 1* (*DOG1*)]. ABI3 is a conserved gene at embryogenesis (Fischerova et al., 2008) and, in association with ARF10/16, regulates seed dormancy (Liu et al., 2013), while DOG1 is involved in dormancy cycling as a response to seasonal environmental signals (Vidigal et al., 2016) and subject to non-coding RNA-mediated mechanisms (Fedak et al., 2016).

A growing body of knowledge reinforces the notion that sRNA dynamics at seed set help shape the capacity of phenotypic variation and local adaptation. Here, we employ an Illumina sequencing approach to identify and comparatively examine enriched sRNAs and their possible targets throughout seed-set phases among populations within and between *P. glauca* and *Arabidopsis*. Through this study, we intend to address three sets of compelling questions: (i) Are top highly expressed miRNAs overlapped in *P. glauca* and *Arabidopsis*? And are they also the deeply conserved ones throughout the plant kingdom? (ii) What are the expression dynamics for lineage- (i.e., sRNAs detected over time across chosen populations) and population-specific sRNAs (i.e., sRNAs detected over time but not across populations)? And are these patterns consistent in the two species? (iii) Are there putative sRNAs targeting known adaptive genes generated at seed development? And how is the role of miRNA-gene interactions at play for our study phenotype (i.e., seed dormancy)? As of December 2016, 494 unique miRNA entities in *Picea* were documented, of which 155 are known on miRBase and 21 are deeply conserved miRNA families in plants (Källman et al., 2013; Xia et al., 2015). Most embryogenesis-related genes in *Arabidopsis* have homologs, with high congruity, in conifers (Cairney and Pullman, 2007), such as in *P. taeda* (83%) (Cairney et al., 2006) and *Larix kaempferi* (78%) (Zhang et al., 2012). The transcriptomic profiling of the zygotic embryo in *P. pinaster* is highly correlated with that in *Arabidopsis* (Xiang et al., 2011; de Vega-Bartol et al., 2013). Their high similarities in gene homologs make possible and meaningful this comparative study between a conifer and Arabidopsis. This study provides us with important clues to further investigating how sRNAs and GRNs coevolve to influence adaptive capacity and organismal diversity.

## Materials and methods

### Plant material, growing conditions, and sample collection

**Figure 1:**
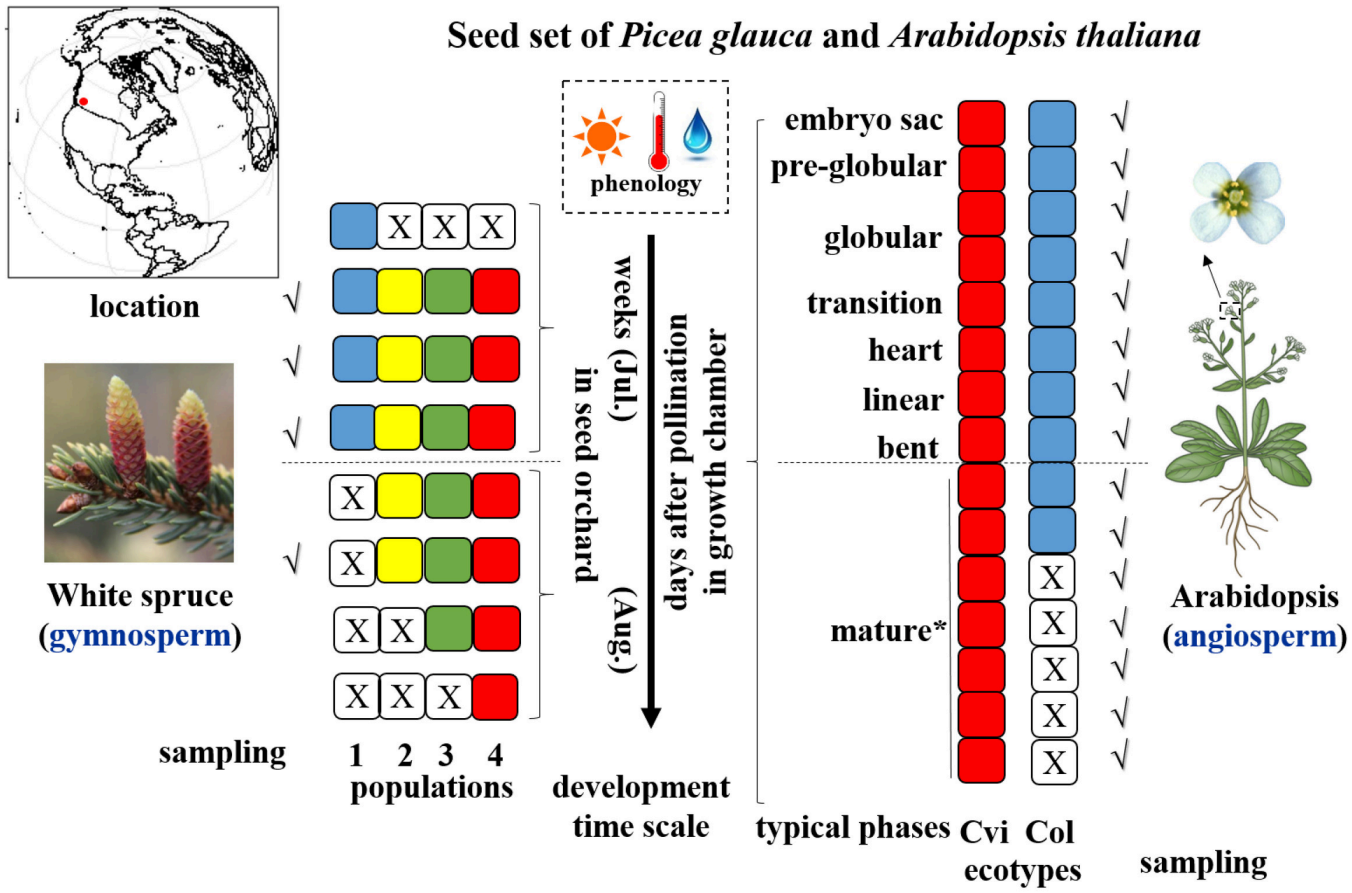
Illustration of sampling strategy during seed set of study species. Different color of square cells represents different populations/ecotypes for *P. glauca* and *Arabidopsis*, where a white square cell with a cross inside represents a corresponding population/ecotype that has not yet been fertilized or has already matured, thus no sampling possibilities in those stages (e.g., only three sampling timepoints for Pop1 of *P. glauca*). Populations of *P. glauca* are exposed to similar phenology during seed set in the seed orchard roughly within weeks of July through August, and ecotypes of *Arabidopsis* are cultivated in three growth chambers with the same growth conditions in periodic cycles to reap three biological replicates. A dashed line in the horizontal direction roughly divides seed development into two phases: morphogenesis (up) and maturation (down). Tick symbols (√) mark the sampling timepoints. An asterisk (^*^) means that after the “mature” phase in *Arabidopsis*, another 5–8 days are needed to complete post-maturation and seed dormancy arrest. Plant images reprinted with permission of source: Google image.

White spruce (*P. glauca*), a keystone species of boreal forests in the North American taiga, is anemophilous (outcrossing) and its seed and pollen cones develop on the same tree and are diclinous (i.e., unisexual). According to seed orchards station records, four populations of *P. glauca* (Pop 1~4), characterized by different pollination timing and seed developmental duration, were chosen and 20 developing cones for each population were collected at early, middle, and late developmental stages for a total of four timepoints at the Kalamalka Research Station seed orchards (50°–51°37′N, 119°16′–120°29′W), British Columbia, Canada (Figure 1). Note that population 1 only had three sampling timepoints due to its short seed developmental span. Accordingly, the climatic data were retrieved from the Station. Likewise, as per contrasting seed-set durations, we selected two wild strains of a model annual selfing plant *A. thaliana*, Cvi-0 and Col-0, originating from the Cape Verde Islands and Columbia (Missouri, USA), respectively. The two *Arabidopsis* ecotypes exhibit contrasting dormancy intensities (Koornneef et al., 2000; Ali-Rachedi et al., 2004). They were cultivated in 2-in planting trays containing soil mixed with slow-release fertilizer 14-14-14 in a growth chamber with 16/8 h day/night photoperiod, PPFD (photosynthetic photon flux density) of 250 μmol·m^2^·s^−1^, and constant temperature of 22°C. Individual flowers were tagged on the day of flowering and developing seeds were sampled manually every day for 9 consecutive days after pollination (DAP) (Figure 1). Five more sampling time points (10–14 DAP) were performed for Cvi-0 due to its slow seed development (Figure 1). Developing seeds at each timepoint were sampled three times (i.e., three biological replicates). After extraction from white spruce cones, direct collection of siliques at Arabidopsis embryogenesis (0–6 DAP) and quick hand-dissection in water from ~30 inflorescences at maturation stages (7 DAP onward), entire developing seeds or siliques were immediately frozen in liquid nitrogen and stored at −80°C until further use. Note that the seed samples from the same developing timepoint were pooled in the same vial for the subsequent analyses.

### RNA isolation, library construction, and sRNA sequencing

Total RNAs were extracted and divided into two aliquots (~15 μg each) from developing seed samples of *P. glauca* and *Arabidopsis* using PureLink Plant RNA Reagent (Ambion) according to the manufacturer's instructions. The intergrity and quantity of the RNA samples were assessed on a BioAnalyser 2100 (Agilent Technologies) and a Nanodrop ND-1000 spectrophotometer (Thermo Fisher Scientific). The sRNA-seq libraries were constructed using a strand-specific and plate-based protocol. To enrich sRNAs, total RNA samples underwent polyA selection using Miltenyi MultiMACS mRNA isolation kit (cat. 130-092-519) following the manufacturer's protocol and the flowthrough (i.e., containing sRNA species without mRNAs) was used for plate-based sRNA construction. A 3′ adapter that is an adenylated single-stranded DNA was selectively ligated to the sRNA template using a truncated T4 RNA ligase 2 (NEB Canada, cat. M0242L). A 5′ adapter was then added using a T4 RNA ligase (Ambion USA, cat. AM2141) and ATPs. After ligation, first strand cDNA was synthesized using a Superscript II Reverse Transcriptase (Invitrogen, cat. 18064 014) and one RT primer. This was the template for the final library PCR, into which 6-nt mers index was introduced to identify libraries (i.e., demultiplexed) from a sequenced pool. Constructed libaries were pooled by phylum; that is, 15 *P. glauca* and 25 *Arabidopsis* samples were pooled seperately, and both lanes were 31 base SET lanes. Sequencing (Illumina HiSeq™ 2500) was implemented using one short SET indexed lane per pool (BC Cancer Agency, Genome Sciences Centre, Vancouver, Canada).

### Small RNA dataset analysis

The sequence data were partitioned into individual libraries based on the index read sequences, and the reads underwent an initial QC assessment. After being preprocessed to clean reads by trimming adapters and barcode sequences using an internal matching algorithm (BC Cancer Agency), the raw sequencing data (in bam format) were converted into sam, fastq, fasta, and txt formats under Linux in a command-line environmemt for subsequent use. The sRNA toolbox was used to profile sRNAs and size distribution, perform conserved miRNA analysis using miRNAs for *Arabidopsis* or a high confidence set of miRNAs on miRBase for *P. glauca*, and their consensus miRNA families and unique sequences (Rueda et al., 2015). sRNAs in sequencing libraries were computationally predicted against the *P. glauca* genome assemblies (highly framented, containing 3.1 million scaffolds longer than 500 bases and a N50 of 43.5 kbp; PG29 v3, 20Gb divided into 30 Mb per file) (Warren et al., 2015) and the Arabidopsis genome (TAIR10), respectively, using miRPlant (An et al., 2014) at default settings with a sequence length cutoff of 18~24 nt. As the size of our *P. glauca* sequence libraries exceeded the maximal single load on miRPlant, we divided each library into several sub-ones with a maxium size of 120 Mb under Linux and after combining the output files in the same library (e.g., summing up raw abundance for the same unique reads from different sub-files), duplicated reads were removed and we only retained one copy of unique reads with the highest prediction score for each library using R 3.2.2 (The R Project for Statistical Computing), followed by a visual inspection to ensure the R coding has attained our objectives.

The libraries substracted r/t/sn/snoRNAs (i.e., ribosomal RNA, transference RNA, small nuclear ribonucleic RNA, and small nucleolar RNA, originally sourced from Sanger RNA family database 12.0, Nawrocki et al., 2015) and non-sRNAs from the total library size, and the resulting number was used for abundance normalization. Abundances were expressed throughout this study in reads per millon (RPM) unless otherwise indicated. Due to the unavailability of complete r/t/sn/snoRNA sequences in *P. glauca*, we utilized Arabidopsis r/t/sn/snoRNA annotations to classify non-sRNAs in *P. glauca*, which include reads of non-predictable secondary RNA structure and non-mapped to the genome. After genome-wide identification of sRNAs, the unique sRNA sequences with raw abundance above 10 at least in one library, along with raw and nomalized counts and precursors, were archived and compared with previously identified spruce sRNAs (Källman et al., 2013; Xia et al., 2015). To isolate conserved miRNAs by using a homolog search, sRNAs in this study were aligned versus a Viridiplantae-specific miRBase reference file containing 100,014 miRNA sequences from 1,397 miRNA families (Chávez Montes et al., 2014), curated in Table S1. Note that miRBase v21 (http://www.mirbase.org/) has archived *c*. 8,000 miRNAs from 73 plant species.

Heat maps graphically depicted the most conserved and differentially expressed miRNAs at seed set across populations/ecotypes, whereby cluster analyses were performed for seed-set phases and key miRNAs within phylum using an euclidean method. Note that raw processed data (i.e., log2 of count per million) underwent a log10-transformation before being fitted on the map.

Top-one targets of the archived unique sRNAs were predicted using transcripts without miRNA genes on psRNATarget (Dai and Zhao, 2011). We focused on the sRNAs of high strength of prediction (score ≥ 0) in *Arabidopsis*, while in *P. glauca*, on sRNAs that were detected in at least 14 of 15 libraries across four populations, termed “most conserved” sRNAs hereafter (N.B. total reads in one library are much less than the others). To annotate target mRNA functions, the top predicted target gene for each sRNA of interest was aligned against the Gene Ontology (GO) protein database for GO term classification and KEGG pathway enrichment (Ashburner et al., 2000; Kanehisa and Goto, 2000).

### Identification of adaptation-associated sRNAs

In a parallel analysis to search for potential sRNAs targeting genes involved in adaptation to climate, we manually refined sRNAs through identifying their target genes that function in stress responses for Arabidopsis. Reportedly, there were 73 key adaptive genes in *P. glauca* (17 overlapped between two articles, Hornoy et al., 2015; Yeaman et al., 2016). We adopted the pipeline of user-submitted small RNAs and transcripts on psRNATarget (Dai and Zhao, 2011) to detect the sRNAs that may target the 73 genes. As most conifer genes were unannotated, we employed a reciprocal BLAST to identify homologs. Specifically, miRNA-targeted genes in *P. glauca* were retrieved via a BLASTN search against the *Arabidopsis* genome on EnsemblPlants (http://plants.ensembl.org) and then putative proteins in *Arabidopsis* were searched via a tBLASTN (six frames) against the *P. glauca* PlantGDB Putative Unique Transcripts (PUTs) database on ConGenIE (http://congenie.org/). Each PUT underwent another BLASTN search against the *Arabidopsis* genome. Homologs were identified only if the same pair of sequences could be found among the top three candidates in the two BLASTNs. Adaptive genes functions were classified based on the records in TAIR10 (https://www.arabidopsis.org/).

### Environment association analysis

To extract expression structures of genotypes (represented by miRNA and mRNA relative expression) that can be explained by environments (temperature at seed set and phenology represented by developmental phase and pattern), redundacy analysis (RDA) was conducted (Oksanen et al., 2015) and visualized by a constrained ordination triplot with both response and explanatory variables in the same coordinate using a scaling 2 method. RDA combines multivariate regression with PCA of multiple dependent variables and we used it to test and quatify the overall contribution of a primary climate variable to the expression pattern of different types of miRNA and mRNAs.This analysis was carried out under R 3.2.2.

### Gene expression analysis

With high confidence, genes targeted by conserved miRNAs of interest were experimentally validated using a quantitative RT-PCR (qRT-PCR) assay as follows. Two microgram of the other aliquot of total RNAs was reverse-transcribed into cDNAs using the EasyScript PlusTM kit (abmGood) with oligo-dT primers following the manufacturer's instructions and first-strand cDNA synthesis products were diluted fivefold as qRT-PCR templates. qRT-PCR was run in 15 μl reaction volumes on an ABI StepOnePlus™ machine (Life Technologies) using the PerfeCTa® SYBR® Green SuperMix with ROX (Quanta Biosciences). The reaction components and procedure were carried out as previously described (Liu et al., 2015). We adopted three technical replicates for each pooled sample of three biological replicates. Reference genes were used as previously descibed (Czechowski et al., 2005; Liu et al., 2015). Gene homolog identifications and primer pairs for the qPCR amplification were listed in Tables S2, S3.

## Results

### Small RNA transcriptomic profiling throughout seed set

**Figure 2:**
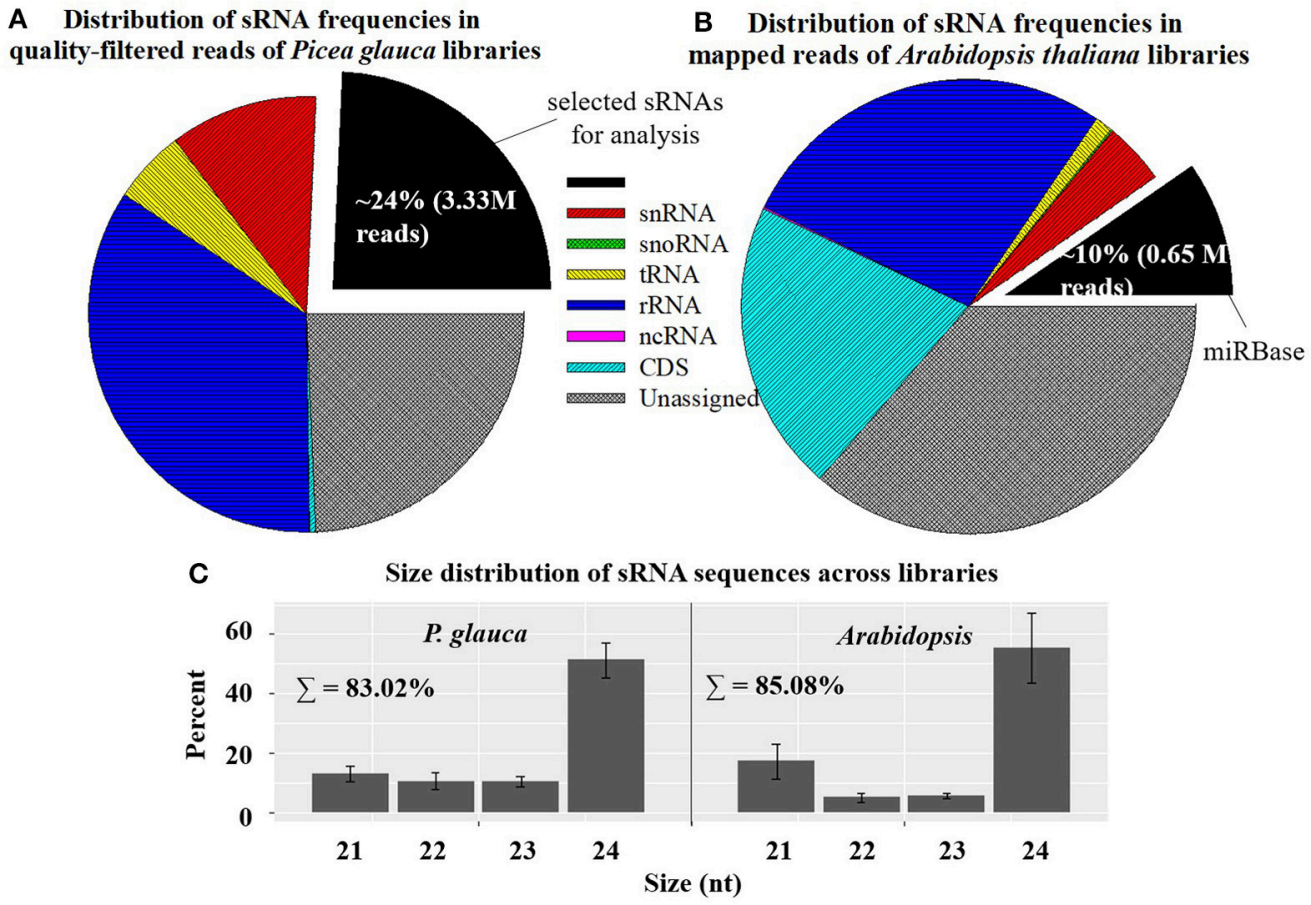
Mapping statistics showing the distribution of sRNA frequencies (by classification; **A,B**) and size 21~24-nt categories; **(C)** in *Picea glauca* and *Arabidopsis thaliana* libraries. Small RNAs mainly include: transfer RNAs (tRNAs), ribosomal RNAs (rRNAs), microRNAs (miRNAs), small interfering RNAs (siRNAs), small nuclear ribonucleic RNAs (snRNAs), small nucleolar RNAs (snoRNAs), long noncoding RNAs (lncRNAs), Piwi interacting RNAs (piRNAs), and repeat associated RNAs (asiRNAs). Relative proportions of sRNA sequences here considered cover the length range from 15 to 30 nt. In genome mapping, miRBase is “sense” (see the legend of pie charts).

Small RNA libraries were prepared from four seed developmental stages of four populations in *P. glauca* (N.B. only three timepoints for population 1 due to its short seed developmental duration) and 10 consecutive days after pollination (0~9 DAP) of Cvi-0 and Col-0 in *Arabidopsis* plus another 5 days (10~14 DAP) for Cvi-0 due to its slow seed development (Figure 1). After a quality filtering, the Illumina deep sequencing yielded 15.1 M reads, on average, in 15 *P. glauca* libraries (Table S4). By contrast, there were on average 10.3 M reads in 25 *A. thaliana* libraries (Table S4). Mapping the quality-filtered reads to their corresponding genome with predictable hairpin RNA secondary structures generated an average of 3.33 M (24 ± 6%) and 7.33 M (84.3 ± 5%) reads in *P. glauca* and *Arabidopsis* libraries, respectively (Figure 2A and Table S4). Low proportion of sRNAs for *P. glauca* may be due to the state of scaffold-level genome assemblies in *P. glauca* compared with the complete assembled genome by chromosomes in the model organism, *Arabidopsis*. Using the archived miRNA reads under *Arabidopsis* species on miRBase, the known miRNAs among quality-filtered sRNA reads occupied on average 0.3% (0.05 M) and 10% (0.65 M) in *P. glauca* populations and *Arabidopsis* ecotypes over time, respectively (Table S4 and Figure 2B). This indicates that an appreciable amount of other types of sRNAs (e.g., siRNAs) is largely produced at seed set of *P. glauca*. The libraries of *Arabidopsis* and *P. glauca* showed, in consistency, that 24-nt sRNAs were dominantly produced at seed set (Figure 2C). In addition, sRNA reads in *P. glauca* classified into r/t/sn/snoRNAs took up 34.4, 5.5, 10.9, and 0.08% of the total sequences, respectively (Figure 2A); analogously, these small RNA categories were 27.3, 1.3, 4.4, and 0.1%, respectively, in *Arabidopsis* (Figure 2B).

**Figure 3:**
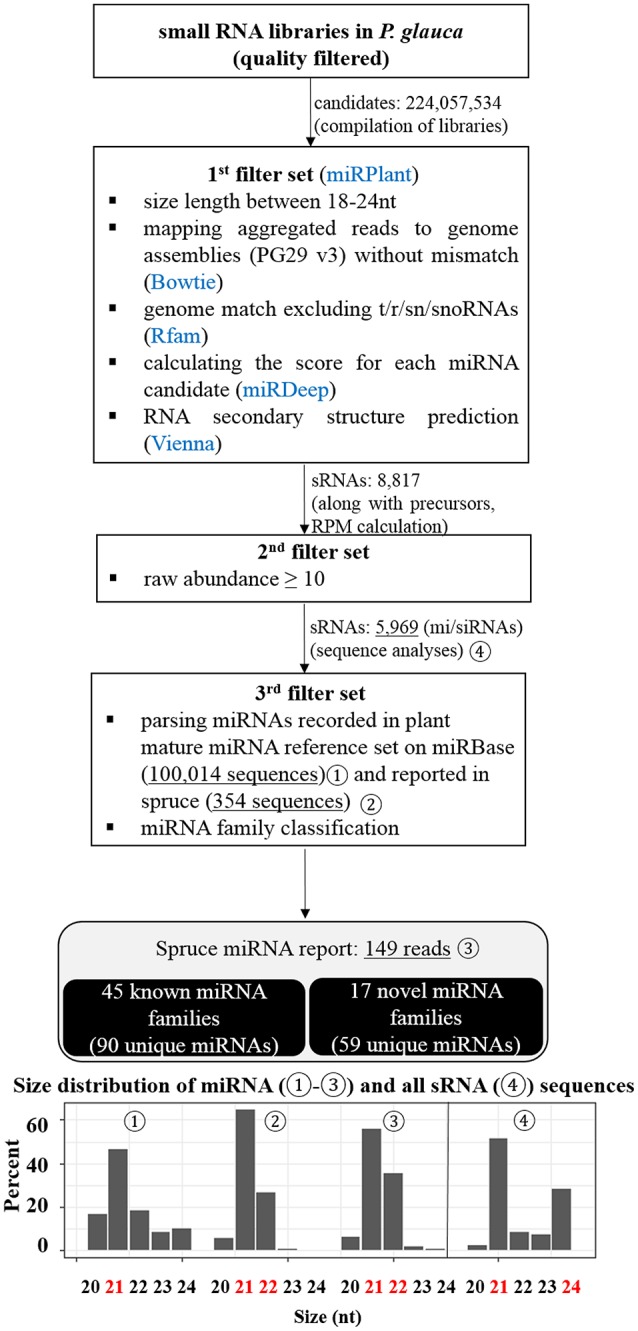
Pipeline for the identification of miRNAs from the small RNA libraries of *P. glauca* and size distribution. miRNAs were identified with adherence to a strict set of filters shown in the diagram and described in the text. After each filter, the number of target candidate sRNAs is indicated and the information of miRNAs is summarized in the last step. Size distribution for four sets of sRNAs (marked in the diagram by circled numbers) is given at the bottom in bar charts.

After a suite of filters for *P. glauca* libraries, we identified 5,969 sRNA sequences, including 149 miRNAs from 62 miRNA families (Figure 3), curated in Tables S5, S6, respectively. Compared with previous reports (Källman et al., 2013; Xia et al., 2015), 90 were known miRNAs, whilst 59 were novel (Table S6). Size distribution showed that miRNAs are mainly 21-nt long throughout the plant kingdom (Figure 3-192) and identified miRNAs in spruce consistently exhibited that 21- and 22-nt were enriched in previous and this study (Figures 3-193,194). As the 21-/22-nt size class mainly consists of miRNA and phasiRNAs (or tasiRNAs), this result suggests that tasiRNAs or phasiRNAs (triggered by miRNA pairing) may play an important role in conifers, as already examined in *P. abies* (Xia et al., 2015). Additionally, the size distribution of all sRNAs showed that both 21- and 24-nt were abundant (Figure 3-195), indicating that hc-siRNAs for the reinforcement of silencing chromatin marks were highly generated typically at seed set in spruce. This observation from developing seeds (~30%) is significantly different from that in buds (~1%) (Källman et al., 2013), which confirms that the 24-nt long sRNA class is specific to reproductive tissues in conifers (Nystedt et al., 2013).

### Association between sRNA expression pattern and seed-set phases

The top 40 conserved and differentially expressed miRNA sequences between different timepoints at seed set were respectively selected from *P. glauca* and *Arabidopsis* libraries and their expression was showcased in bivariate plots (Figure S1). In small panels of Figure S1, histograms along the diagonal were distributed in a right-skewed manner and scatter plots for *P. glauca* and *Arabidopsis* generally showed a monotonic and linear relationship, respectively. This indicates that the expression of conserved miRNAs is intrinsically correlated among different phases of seed set and populations/ecotypes.

**Figure 4:**
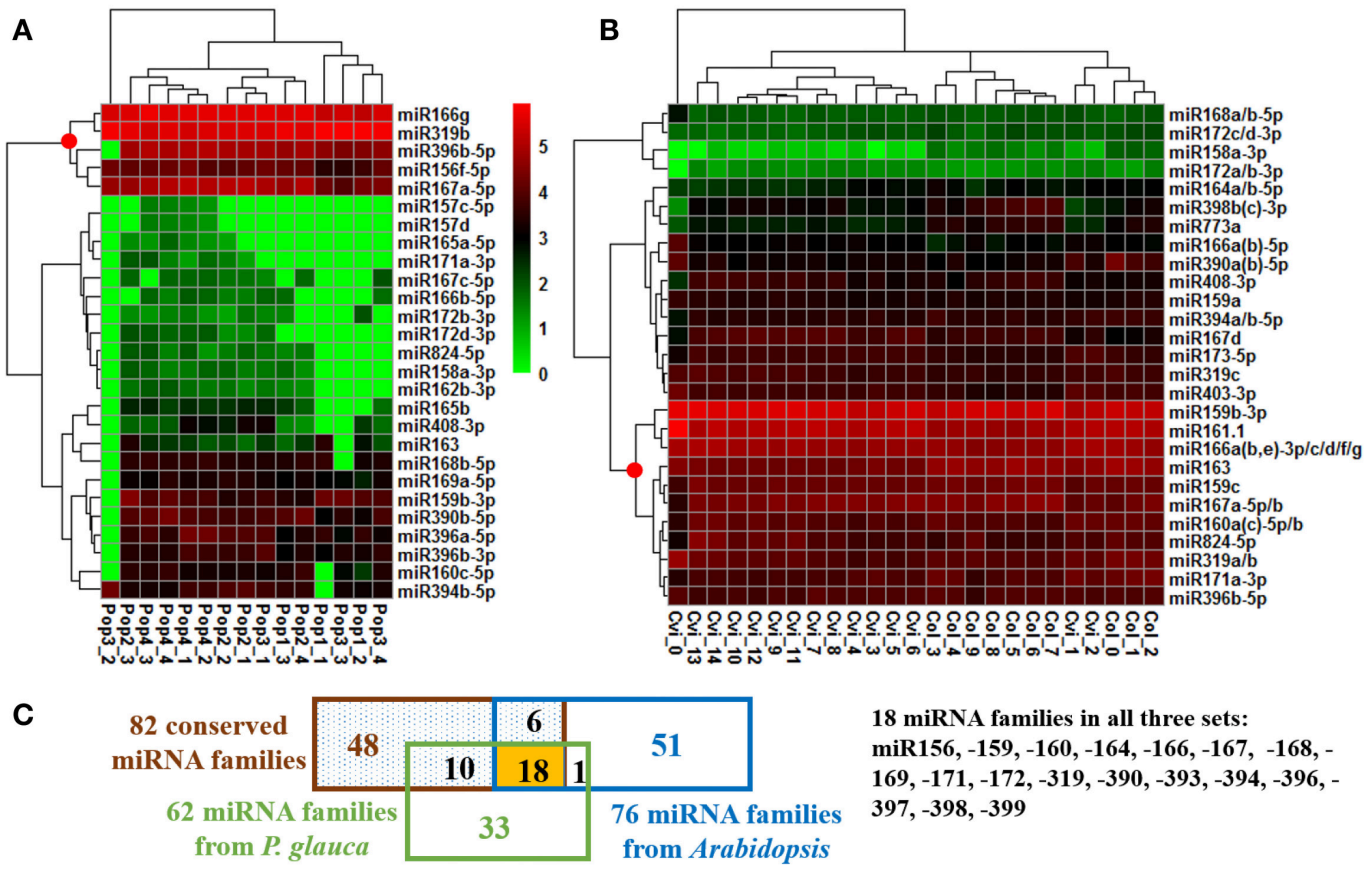
Temporal expression pattern of top conserved miRNAs in *P. glauca* and *Arabidopsis*. We used hierarchical clustering of 27 most abundant conserved miRNAs and seed developmental phases in **(A)** *P. glauca* and **(B)** Arabidopsis. The color key alludes to the relative expression level: green- low, black- medium and red- high corresponding to the ranges (log2-transformed RPM) of [0, 625000] and [0, 536276] for *P. glauca* and *Arabidopsis*, respectively. A red dot at the node for miRNA clustering indicates highly expressed miRNAs in *P. glauca* and *Arabidopsis*, respectively. An underscore line links population number to *P. glauca* seed-set phase (e.g., “Pop1_1” means Population 1 at seed-set timepoint 1, and so forth) or ecotype to *Arabidopsis* seed-set phase (e.g., “Cvi_1” denotes Cvi-0 at timepoint 1, and so forth). In **(C)** miRNA family Venn diagram, the “82 conserved miRNA families” across Tracheophyta species are from Chávez Montes et al. (2014).

The expression of the top 27 conserved miRNA families in *P. glauca* and *Arabidopsis* seed set was respectively employed to create a heat map and perform cluster analyses for seed-set phases and normalized miRNA expressions over time (Figures 4A,B). In *P. glauca*, the seed-set phases in the same population were not completely classified in one of the four major “clades” (Figure 4A). However, in *Arabidopsis*, the phases at 3DAP onward were neatly clustered into two primary groups by ecotype (Figure 4B). The expression pattern for Cvi-0 at flowering was different from other phases and it constituted a single “clade” (Figure 4B), while early seed-set for Cvi-0 (i.e., Cvi_1~2) and Col-0 (i.e., Col_0~2) were within the same group (Figure 4B). This indicates that the pattern of relative expression of conserved miRNAs was highly correlated with seed-set phases in *Arabidopsis* but not in *P. glauca*, and miRNAs significantly regulate seed development at least as early as 3DAP in globular embryos of Arabidopsis ecotypes. Regarding the most highly expressed and conserved miRNAs, miR166g and −319b were most abundant, followed by miR396b-5p, −156f-5p, and −167a-5p, in *P. glauca* (Figure 4A), while in *Arabidopsis*, most enriched ones were miR159b-3p, −161.1, −166a(b,e)−3p/c/d/f/g, −163, and −159c, 167a-5p/b (Figure 4B). Taken together, miR166, −319, and −396 families were highly expressed in both *P. glauca* and *Arabidopsis* and reportedly, they play regulative roles in seed/cell development (see a summary for selected miRNAs and their functions in Table S8). In general, the most abundant miRNA families (under the “clade” marked by a red dot in Figure 4) were overlapped in *P. glauca* and *Arabidopsis* but abundant miRNA families were less in number in *P. glauca* (Figure 4). Moreover, 18 conserved miRNA families across vascular plants were identified in both *P. glauca* and *Arabidopsis* (Figure 4C), in which some were engaged in auxin and GA signaling pathways, including miR159, −160, and −167 (Figure 4 and Table 1). In addition, there were conserved miRNAs uniquely detected at seed set in the Col-0 ecotype or in the population of late maturation (Pop 4) in *P. glauca* (Table 2).

**Table 1:** Identification of conserved miRNA isoforms identified in both *Arabidopsis* and *P. glauca* during seed set.

**Name^a^**	**Predicted target^b^**		**Alignment**	**Target gene function^c^**	**E^d^**	**UPE^e^**
miR159b-3p	AT1G18080.1	miRNA	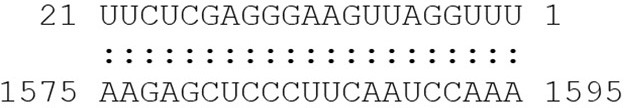	GA and flowering pathways	0	22
		Target				
	BT123375	miRNA	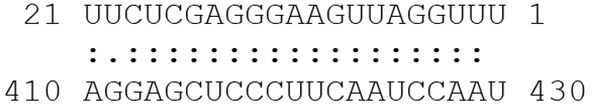	–	1.5	12
		Target				
miR160c-5p	AT1G77850 AT2G28350 AT4G30080	miRNA	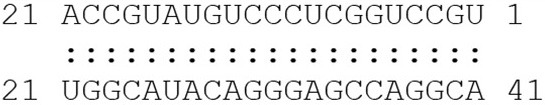	AUXIN RESPONSE FACTOR (ARF) 10, 16 and 17	0	17
		Target				
	BT119832	miRNA	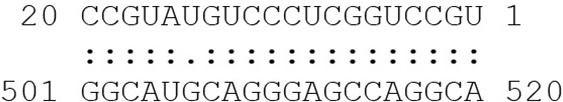	Putative ARF 10/16/17 (NCBI No. FN433183) in *Cycas rumphii*	0.8	19
		Target				
miR163	AT1G66720.1	miRNA	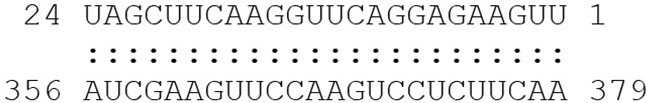	Methylation	0	13
		Target				
	BT112171	miRNA	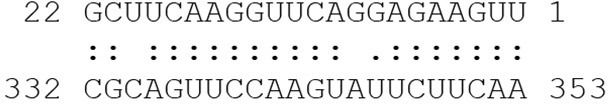	–	2.5	15
		Target				
miR166b-5p	AT4G14713.1	miRNA	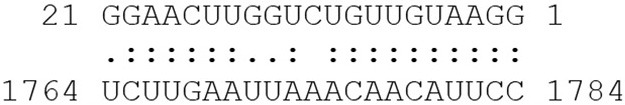	Cell proliferation	2	13
		Target				
	EF677221	miRNA	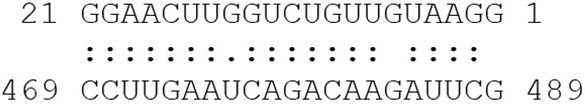	–	3	15
		Target				
miR166g	AT2G34710	miRNA	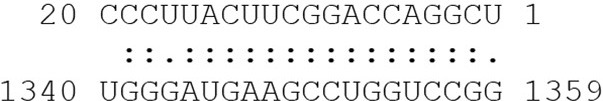	HOMEOBOX PROTEIN 14, associated with development	2	21
		Target				
	HQ391915	miRNA	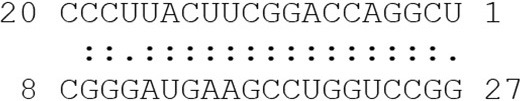	Homeodomain leucine zipper protein	2	16
		Target				
miR167a-5p	AT1G30330 AT5G37020	miRNA	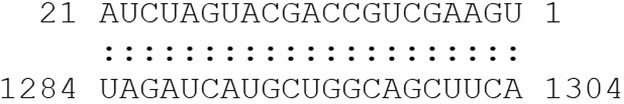	AUXIN RESPONSE FACTOR 6 and 8	0	24
		Target				
	FJ469921	miRNA	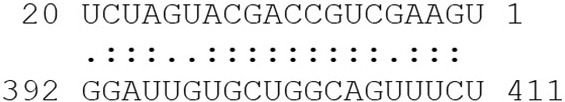	R2R3-MYB transcription factor	3	18
		Target				
miR171a-3p	AT3G60630.1	miRNA	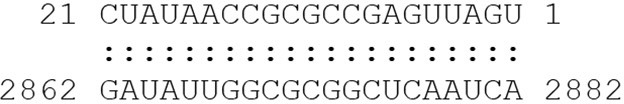	Cell differentiation and division	0	14
		Target				
	BT102743	miRNA	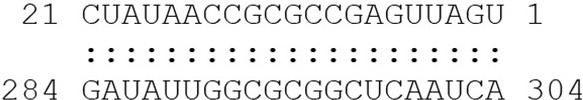	–	0	17
		Target				
miR319b	AT4G23710.1	miRNA	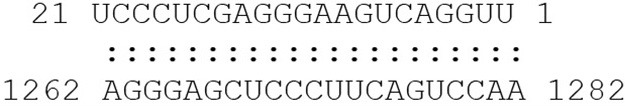	Proton transport	0	15
		Target				
	BT110042.1	miRNA	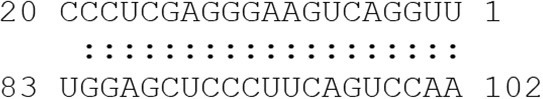	–	1	22
		Target				
miR390b-5p	AT5G03650.1	miRNA	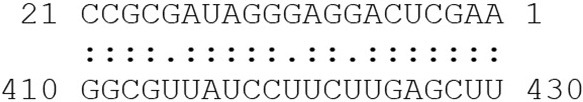	Starch branching enzyme	1.5	21
		Target				
	EX354481	miRNA	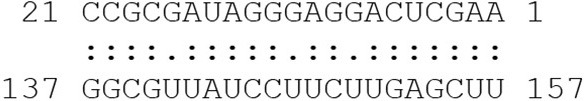	–	1.5	17
		Target				
miR394b-5p	AT1G27350	miRNA	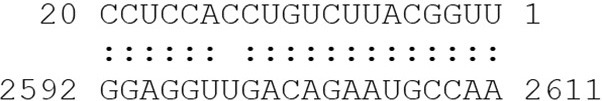	Ribosome associated membrane protein	1	15
		Target				
	BT112917	miRNA	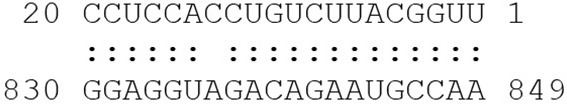	–	1	20
		Target				
miR396-5p	AT1G53910	miRNA	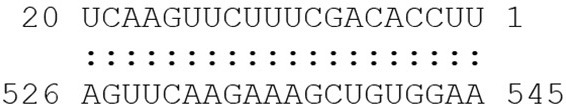	Ethylene response factor	0	14
		Target				
	BT102125	miRNA	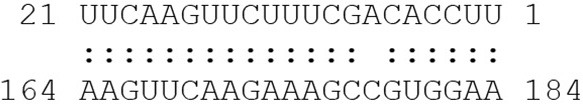	–	1.5	34
		Target				
miR408-3p	AT2G02860	miRNA	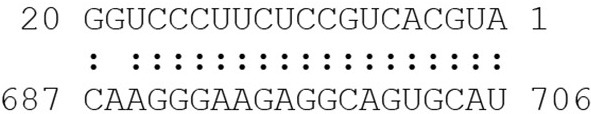	SUCROSE TRANSPORTER 3	1	23
		Target				
	BT103532	miRNA	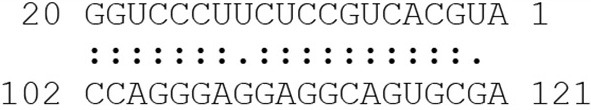	–	2	25
		Target				
miR824-5p	AT3G57230	miRNA	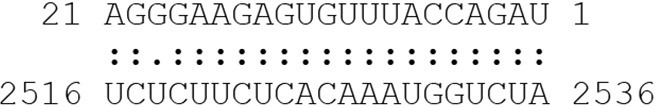	MADS-box transcription factor	0.5	15
		Target				
	BT112142	miRNA	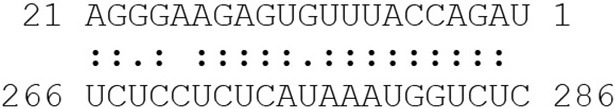	–	3	13
		Target				

a *Nomenclature of miR: (organism)miRnx - precursor arm and/ or.y, where n, a sequential number representing family of miR; x, lettered suffixes representing family member (i.e., closely related mature sequences); −5p or −3p denote 5′ or 3′ arm of the precursor;.y, integer denoting occurrence of more than one mature sequence from the same precursor*.

b *For each miRNA, shown is the most confidently predicted in Arabidopsis (above) and P. glauca (below)*.

c *Refer to GO enrichment analysis, TAIR10 and NCBI*.

d *Expectation (E), stringent threshold [0–0.2] gives lower false positive prediction*.

e *Maximum energy to unpair the target site (UPE), small value (range ϵ [0, 100]) is better*.

**Table 2:** Identification of unique and conserved miRNAs in Arabidopsis ecotype Col-0 compared with Cvi-0 and studied *P. glauca* population Pop 4 compared with Pop1~3 during seed set.

**Name**	**Predicted target**		**Alignment**	**Target gene function**	**E**	**UPE**
***Arabidopsis thaliana***						
ath-miR158b	AT3G10740.1	miRNA	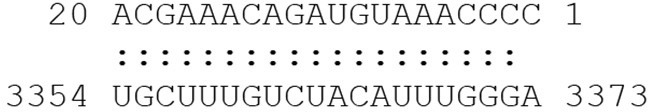	Xylan metabolism (cell wall modification)	1	19
		Target				
ath-miR447c-5p	AT4G03440.1	miRNA	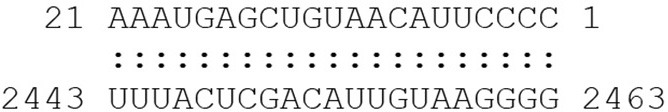	Ankyrin repeat family protein^¶^ (protein-protein interaction)	0	18
		Target				
ath-miR774a	AT3G19890.1	miRNA	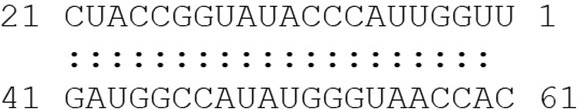	F-box protein	1	19
		Target				
ath-miR776	AT1G08760.1	miRNA	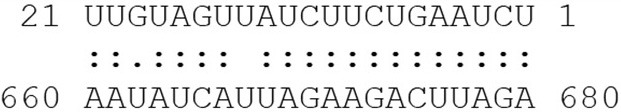	Unknown	1.5	7
		Target				
ath-miR779.1	AT2G22500.1	miRNA	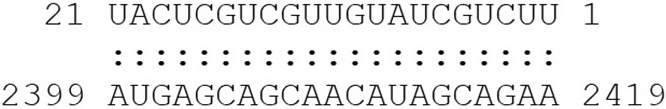	Mitochondrial dicarboxylate carriers (proton transport)	0	12
		Target				
ath-miR832-3p	CP002687	miRNA	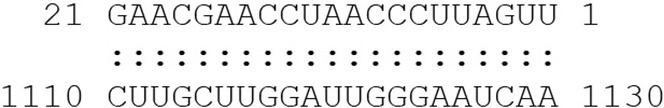	Intergenic, chr. 4	0	13
		Target				
ath-miR833a-5p	CP002687	miRNA	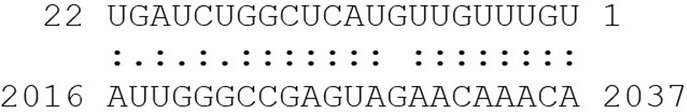	Intergenic, chr. 4 centromere region	2	16
		Target				
ath-miR843	AT3G13840.1	miRNA	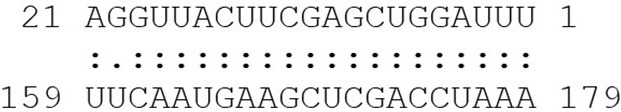	GRAS family transcription factor (regulation of transcription)	0.5	18
		Target				
ath-miR860	CP002684	miRNA	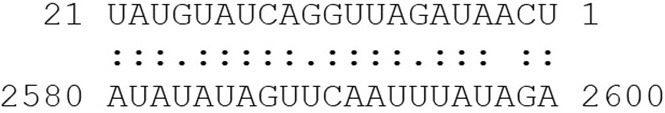	Intergenic, chr. 1	3	14
		Target				
ath-miR864-5p	AT3G11080.1	miRNA	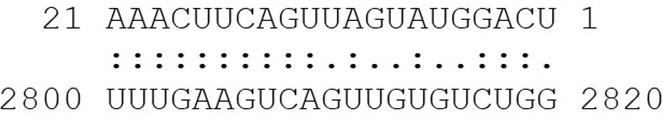	Receptor-like protein 35, signal transduction	3	16
		Target				
ath-miR1886.2	AT2G37160.1	miRNA	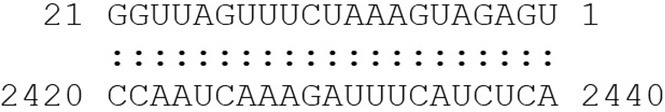	Transducin/WD40 repeat-like superfamily protein	0	30
		Target				
ath-miR3440b-5p^†^	AT5G08490.1	miRNA	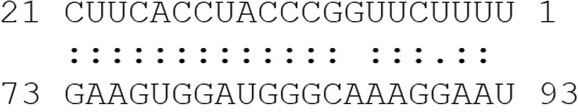	Response to ABA	2.5	23
		Target				
ath-miR5026	CP002688	miRNA	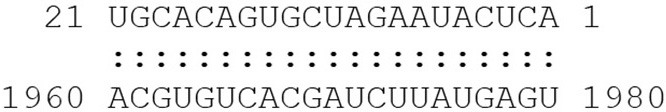	Intergenic, chr. 5	0	15
		Target				
ath-miR5644	AT5G41620.1	miRNA	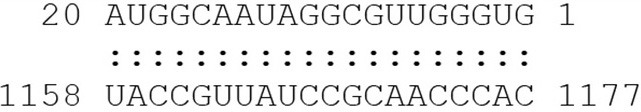	Cell morphogenesis	0	17
		Target				
ath-miR8169	AT3G24340.1	miRNA	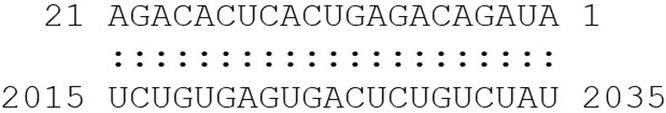	Chromatin remodeling 40	0	15
		Target				
ath-miR8171	AT5G56380.1	miRNA	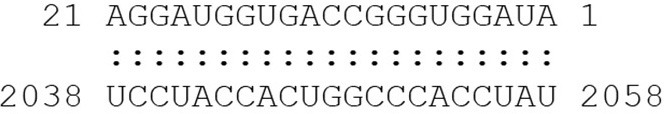	F-box protein	0	21
		Target				
***Picea glauca***						
pgl-miR157c-5p	BT105462	miRNA	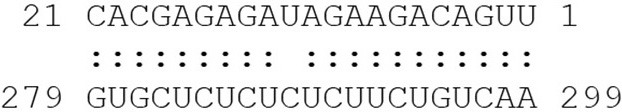	Male and female cone development in *Pinus* (*PtSPL1*, KJ711108)	1	23
		Target				
pgl-miR157d	BT119207	miRNA	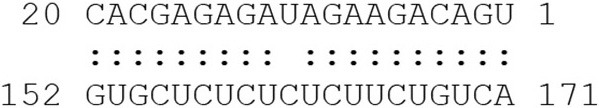	– (mRNA seq)	1	18
		Target				

*The header notation is identical with Table 1*.

† *comPARE predicts that its validated target is AT3G01460, which is involved in embryo development ending in seed dormancy*.

¶ *ANK gene cluster is consistent with a tandem gene duplication and birth-and-death process*.

**Figure 5:**
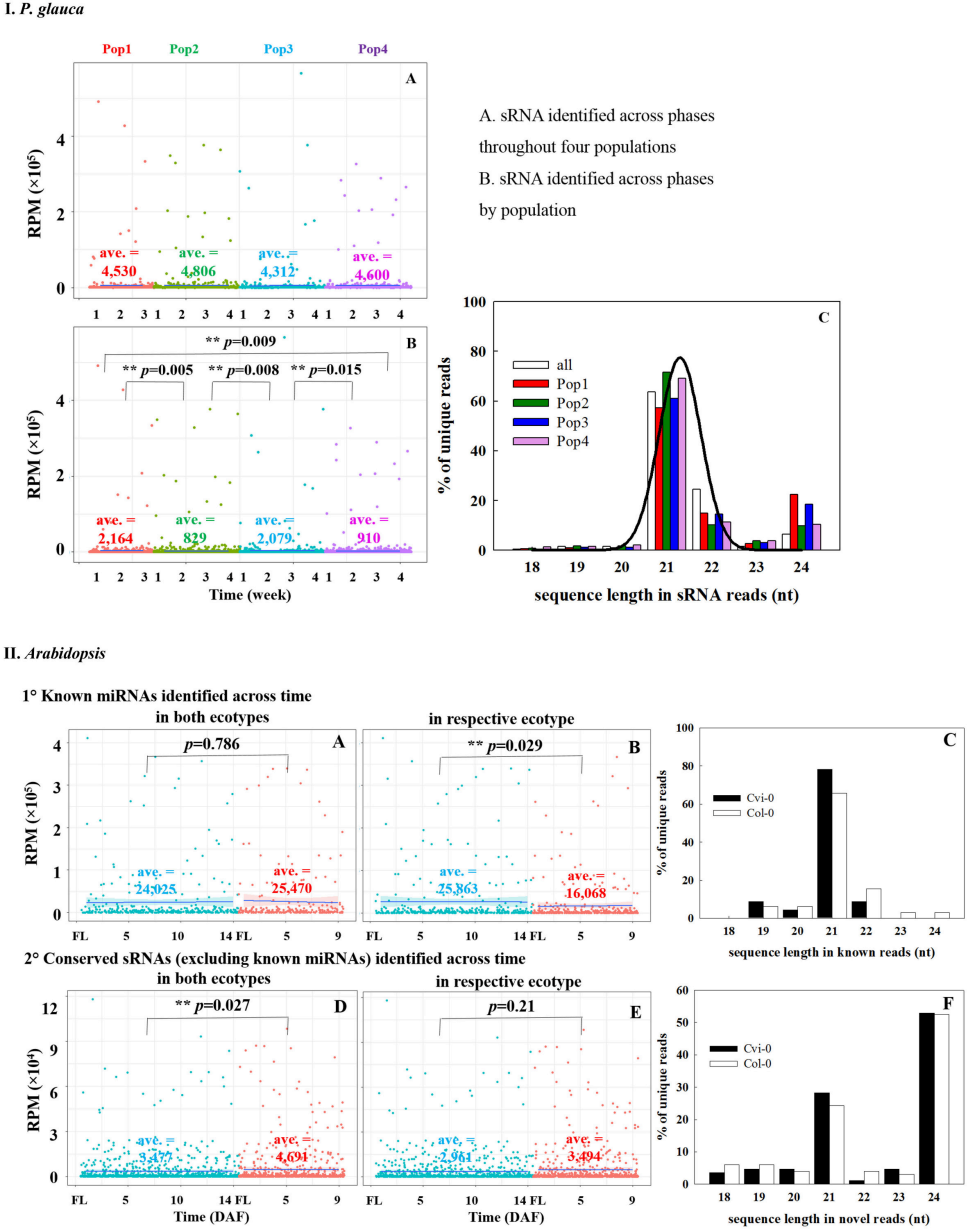
Comparison of expression abundance and pattern of sRNAs identified across seed developmental phases throughout or by populations in *P. glauca* **(IA–C)** and *Arabidopsis* **(IIA–F)**. The normalized average expression is given within each panel (i.e., ave.). ^**^p-value < α (= 0.05) using Student's *t*-test.

**Figure 6:**
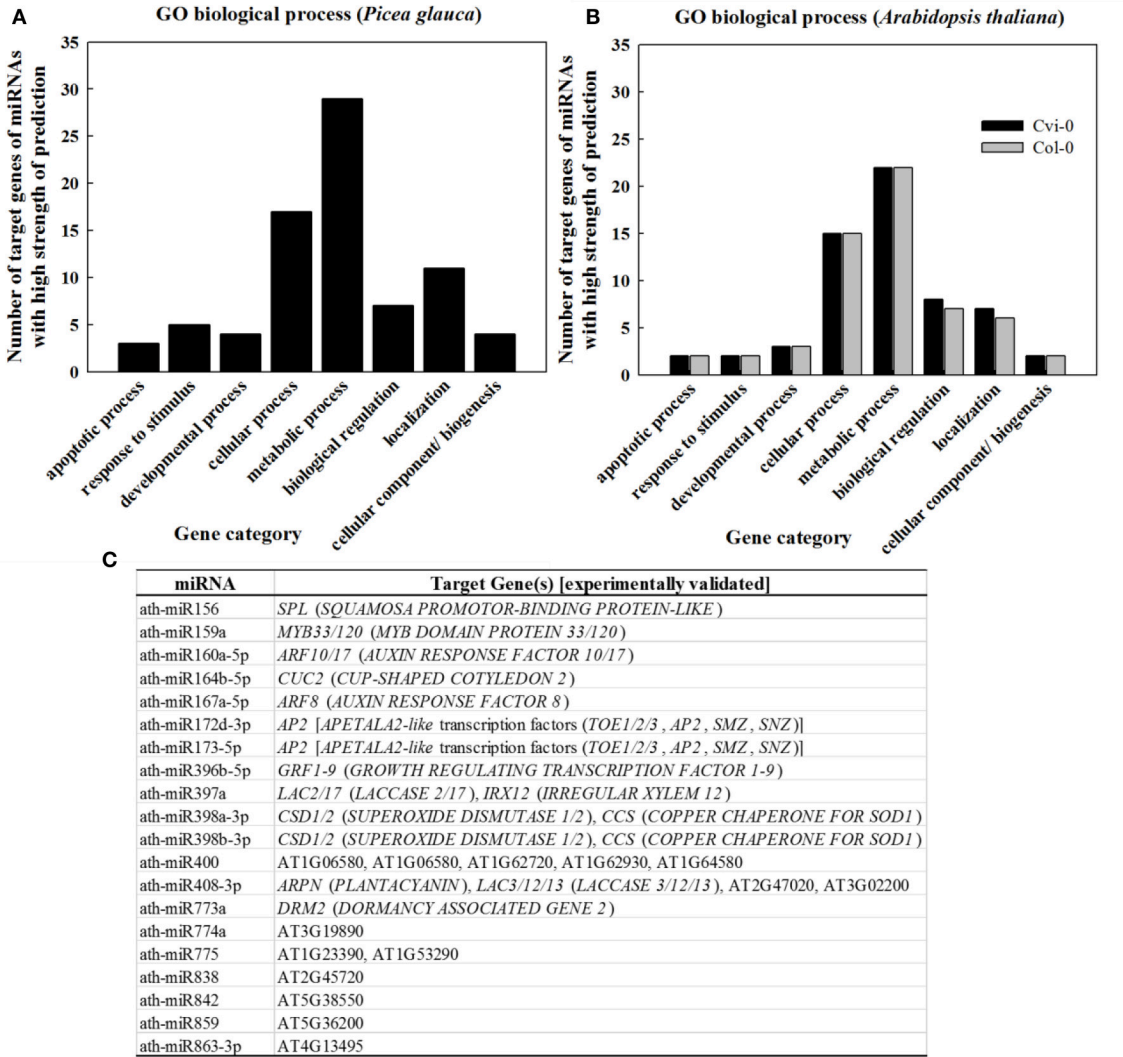
GO classification as per biological process for genes targeted by sRNAs of high strength of prediction **(A,B)** and **(C)** experimentally validated ath-miRNA-target interactions. Gene category and GO code are apoptotic process (GO:0006915), response to stimulus (GO:0050896), developmental process (GO:0032502), cellular process (GO:0009987), metabolic process (GO:0008152), biological regulation (GO:0065007), cellular component organization or biogenesis (GO:0071840), and localization (GO:0051179). Source for validated ath-miRNA-target interactions: miRTarBase (Chou et al., 2016) and previous studies summarized in Table S8.

**Figure 7:**
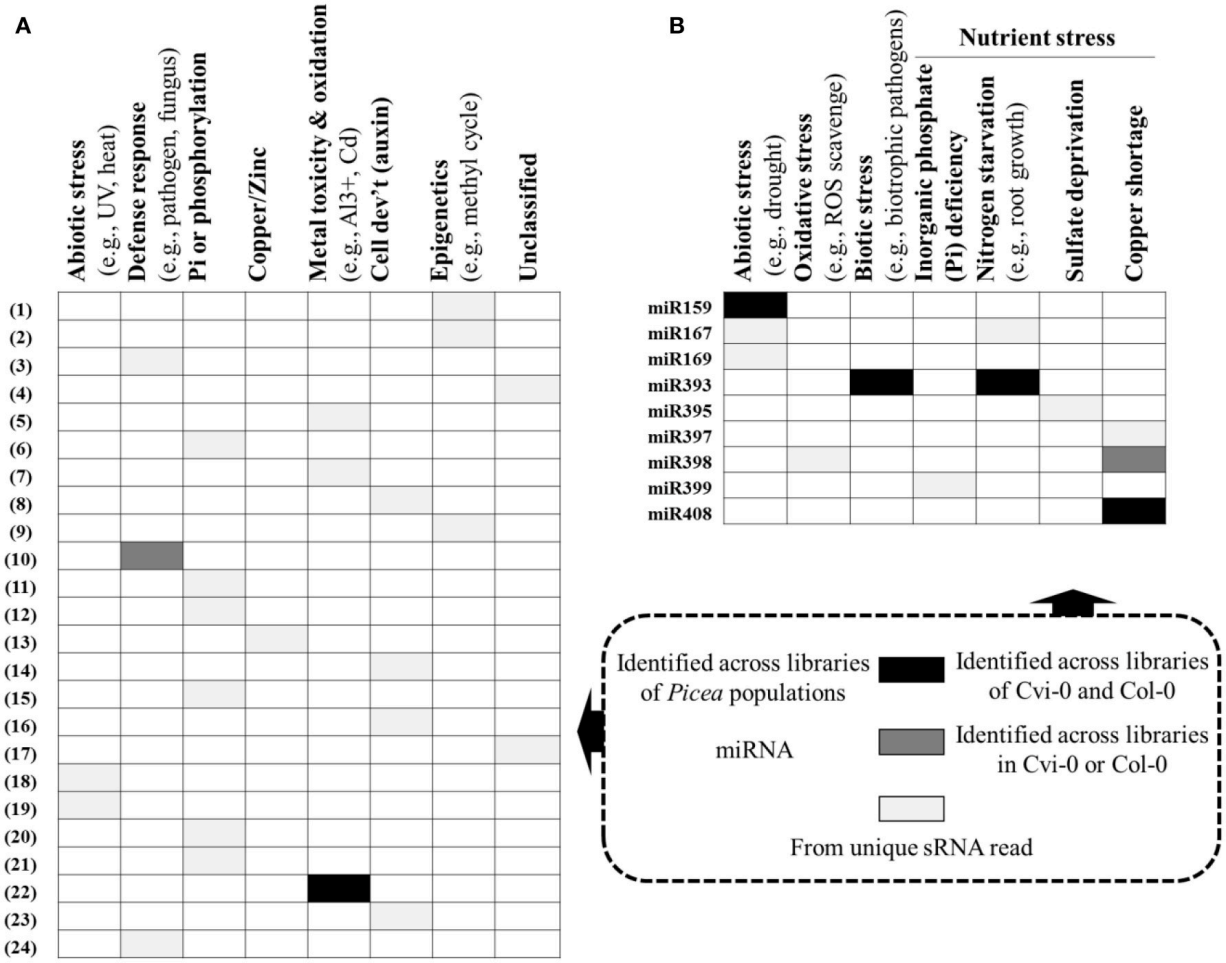
Potential sRNAs targeting adaptive genes in *P. glauca* **(A)** and *Arabidopsis* **(B)**. Legends for white/gray/black cells are given in the rectangular square, bounded in dashed lines. More information about sRNAs in **(A)** is given in Table 3 (N.B. the numerical numbers in **(A)** correspond to the row marks in Table 3). Reported miRs involved in Arabidopsis adaptability: miR159 (Reyes and Chua, 2007), miR167 (Kinoshita et al., 2012), miR169 (Li et al., 2008), miR393 (Navarro et al., 2006, 2008; Vidal et al., 2010; Zhang et al., 2011), miR395 (Jones-Rhoades and Bartel, 2004; Kawashima et al., 2009; Jagadeeswaran et al., 2014), miR397 (Abdel-Ghany and Pilon, 2008; Dong and Pei, 2014), miR398 (Abdel-Ghany and Pilon, 2008; Sunkar et al., 2012; Brousse et al., 2014), miR399 (Aung et al., 2006; Bari et al., 2006), and miR408 (Abdel-Ghany and Pilon, 2008). Because the sRNA expression pattern can be species- and even genotype-specific under stresses [e.g., different expression pattern of miR171 in cold-tolerant and cold-sensitive cultivars of *Camellia sinensis*, (Zhang et al., 2014)], we do not summarize up- or down-regulations of the sRNAs under stresses.

Because the majority of miRNA families has a very limited taxonomic distribution (Cuperus et al., 2011), it is meaningful to carry out a lineage- and population-specific sRNA analysis. Here, lineage-specific sRNAs were represented by sRNAs identified throughout the studied populations in respective species. In *P. glauca*, there was no significant difference in expression abundance and pattern for lineage-specific sRNAs (*p* > 0.05; a total of 187 sequences used) (Figure 5IA). However, the expression pattern was significantly different between populations, when population-specific sRNAs (435, 1181, 433, and 1065 sequences for Pop 1~4, respectively) were used for comparison (all *p*-values < 0.05) (Figure 5IB). The sequence length peaked only in the 21-nt size class (no longer at 24-nt size; compare Figures 3-195, 5IC), supportive to miRNAs and/or tasiRNAs that were abundantly generated and may be selected for.

Analogously, there were 91 conserved miRNAs identified from sequence libraries (Table S9), in which 85 were expressed in both early (Cvi_0~7 and Col_0~4) and late (Cvi_8~14 and Col_5~9) seed-set phases (Figure S3). Conserved ath-miRNAs had a dominant length of 21 nt (Figure 5IIC) and there was no significant difference in the expression pattern for known ath-miRNAs identified across the seed-set period in both ecotypes (*p* = 0.786; totalizing 23 sequences used) (Figure 5IIA), while significantly different when known ath-miRNAs in either ecotype were compared (*p* = 0.029; 23 and 47 sequences for Cvi-0 and Col-0, respectively) (Figure 5IIB). Interestingly, there was a significant difference for sRNAs in both ecotypes (*p* = 0.027; totalizing 69 sequences; known ath-miRNAs excluded) (Figure 5IID) and no significant difference was found for the ones in either ecotype (*p* = 0.21; 85 and 99 sRNAs for Cvi-0 and Col-0, respectively) (Figure 5IIE). The size of these sRNAs peaked at the 21- and 24-nt size classes (Figure 5IIF). This indicates that hc-siRNAs, as well as tasiRNAs and novel miRNAs may have undergone selection in *Arabidopsis*.

### Small RNA frequent emergence and demise throughout time

In *Picea glauca*, considerable sRNAs were generated across seed-set phases in populations (Figure S2). The number of sRNAs detected in all four populations (1,318 reads) was as many as that of unique sRNAs in different populations (1,200 reads on average) (Figure S2A). The unique sRNAs that were expressed across developing phases in all populations were less in number than those that were only detected in one single population (Figure S2B). Burgeoning sets of sRNAs are analogous to the frequent emergence and decay previously reported in novel miRNAs in *Arabidopsis* (Fahlgren et al., 2007). In *Arabidopsis*, the number of sRNA reads without conserved miRNAs was almost 10 times as many as conserved miRNA sequences (Figure S3A). Different from conserved miRNAs that were expressed in both early and late seed-set phases, these unknown sRNAs were differently produced in phases as well as between ecotypes (Figures S3A,B). A total of 705 unknown sRNA reads out of 5,749 reads were detected in both early and late seed-set phases in the two ecotypes (Cvi_0-7 vs. Cvi_8-14 and Col_0-4 vs. Col_5-9; Figure S3B), in which 44 sequences were expressed across all seed-set phases in two ecotypes.

### Targets of abundant sRNAs and their involvement in adaptation

In light of the tight correlation between sRNA conservation and expression abundance (Chávez Montes et al., 2014), GO classification for target genes of the 107 “most conserved” sRNAs in *P. glauca* (Table S7) showed that sRNAs were mostly involved in the regulation of pathways related with metabolic and cellular processes (Figure 6A), which was in line with the observation in conserved miRNAs (Table S9) in *Arabidopsis* (Figure 6B). The experimentally validated miRNA-gene pairs were listed in Figure 6C and their target genes are mainly involved in seed development (Figure 6C).

We adopted an Illumina sequencing approach to get a deep coverage of mature sRNAs and predicted sRNA candidates that may target the reported genes involved in adaptation to climate in *P. glauca*. We found 24 sRNA candidates (Table 3) targeting genes putatively functioning mainly in abiotic and biotic stresses (Figure 7A). In model organisms, a group of key and conserved miRNA sequences involved in plant seed development and phase transitions was summarized in Table S8, some of which are involved in adaptation to stresses (see description in the “function” column). They were identified by fold changes in miRNAs between stress-treated and control samples. With reference to previous reports, we identified 9 miRNAs involving in adaptation during *Arabidopsis* seed development and they were miR159, −167, −169, −393, −395, −397, −398, −399, and −408 (Figure 7B). These miRNA families also have functionalities in seed development via regulating transcriptional factors in hormone-based GRNs, such as miR159, −167, −393 targeting *GAMYB, AUXIN RESPONSE FACTORS* (*ARF*s), auxin-receptors and cyclin-like F-box, respectively (see a summary in Table S8).

**Table 3:** Potential sRNAs targeting genes responsible for adaptation to climate in *P. glauca*.

**Mature_miR**		**Alignment**	**E**	**UPE**	**Inhibition**	**Clone_ID**	**GeneBank Acc**.	**PUT-175a-Picea_glauca**	**TAIR_ID**
(1) aaaaaggagagttgcctgtgg	miRNA	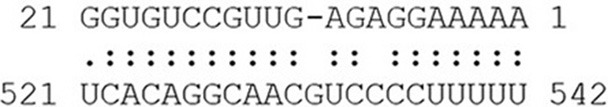	3	6.517	Translation	GQ03005_C08	GT738895	46326	AT2G06990
	Target								
(2) aaacgtctggacgaggtaggctct	miRNA	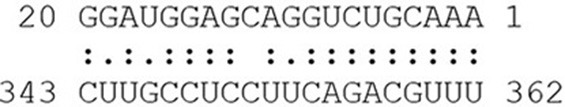	2.5	5.91	Cleavage	GQ03503_O12	GR222863	39402	AT5G66180
	Target								
(3) aaagtcccgaaggcatttgga	miRNA	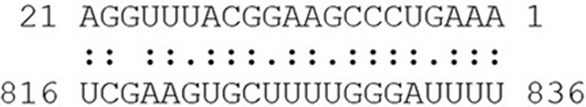	3	18.532	Cleavage	GQ04004_N24	BT118305	32392	AT1G26550
	Target								
(4) aagattttggtttgactagtagag	miRNA	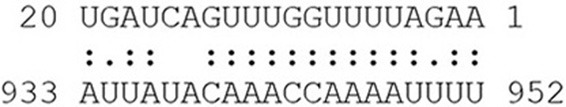	3	10.313	Cleavage	GQ04006_H22	EX437907	9863	AT1G44910
	Target								
(5) aaggttttgttgatttttggg	miRNA	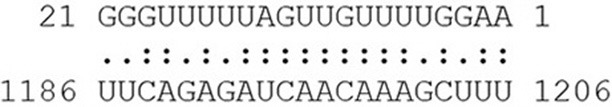	2.5	17.065	Cleavage	GQ0192_K18	BT102435	29740	AT1G64710
	Target								
(6) aatggtttgtgctgagaagatc	miRNA	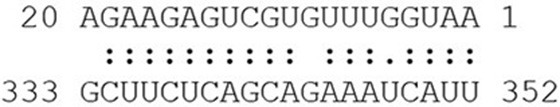	2.5	15.302	Translation	GQ0198_L08	BT102638	16037	AT5G01920
	Target								
(7) actttataaagacttgactgg	miRNA	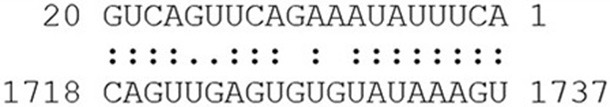	3	11.618	Translation	GQ04109_B04	BT119709	44994	AT2G29550
	Target								
(8) agcttgtataccagtttgtggaca	miRNA	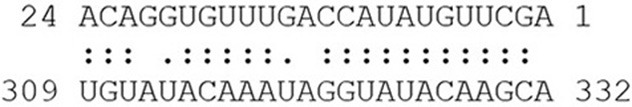	3	24.121	Cleavage	GQ03104_J01	EX347533	33342	AT5G60880
	Target								
(9) agggaagaaaggaaaagaaggggc	miRNA	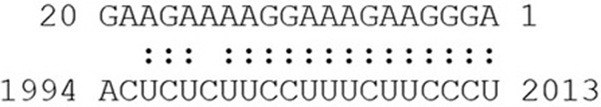	3	15.913	Cleavage	GQ04111_G11	BT119867	49070	AT3G59970
	Target								
(10) atcggggaagttgaatttggc	miRNA	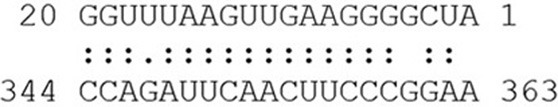	3	15.682	Cleavage	GQ03804_G08	BT116671	42882	AT1G02170
	Target								
(11) atgagatgtgttcaggctgta	miRNA	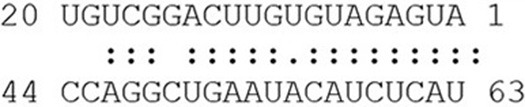	2.5	20.418	Cleavage	GQ02811_J12	BT104537	19871	AT1G10430
	Target								
(12) atgattggtgaagaacttgaaccc	miRNA	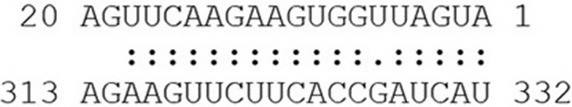	2.5	20.438	Cleavage	GQ03108_B05	BT107479	25696	AT3G19100
	Target								
(13) attcctcaccagatttcgggcaaa	miRNA	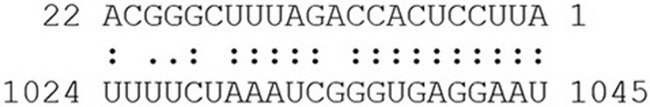	3	16.728	Translation	GQ0041_H19	BT100742	2049263	AT1G08830
	Target								
(14) taacttcgtcggatattcaccatt	miRNA	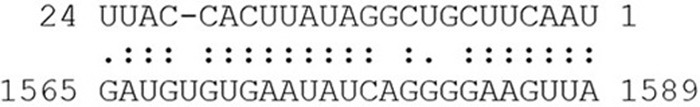	2.5	23.545	Translation	GQ02805_P05	BT104058	39309	AT1G21410
	Target								
(15) tattgatcagctggatgtatt	miRNA	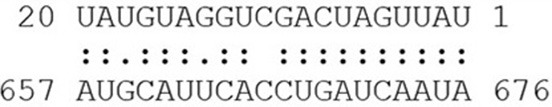	2	13.18	Translation	GQ03235_A09	GO363013	42706	AT2G40270
	Target								
(16) tatttgaagtcggagacctga	miRNA	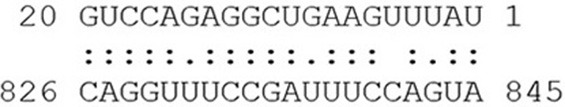	3	12.093	Cleavage	GQ03204_B14	BT109051	38812	AT2G46690
	Target								
(17) tctcttcttttatgcattctag	miRNA	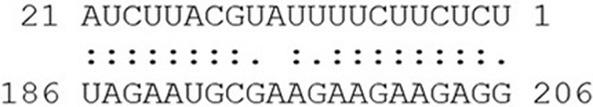	2.5	13.816	Cleavage	GQ02820_B08	BT105240	49160	AT5G22090
	Target								
(18) tcttccaaacataccaatgcg	miRNA	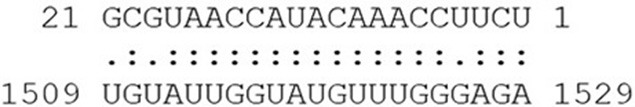	1	22.508	Cleavage	GQ02512_H19	BT103401	42372	AT5G26680
	Target								
(19) tgggcgtttggtgataatatc	miRNA	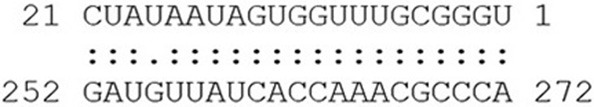	0.5	12.239	Cleavage	GQ0254_G19	BT103470	30277	AT1G09080
	Target								
(20) ttaaagtcgttgaagttgtgt	miRNA	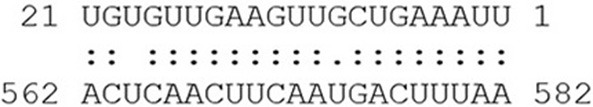	1.5	17.554	Cleavage	GQ03204_I10	GT739039	24064	AT5G62310
	Target								
(21) ttctctttccattgttatcgg	miRNA	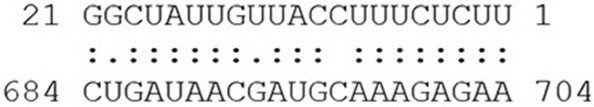	2	10.58	Translation	comp1174_c0^*^	NA	27553/31924	AT5G19760
	Target								
(22) ttcgttggactgtatgctggc	miRNA	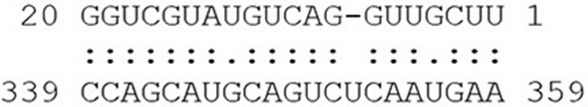	3	12.467	Cleavage	GQ03811_D01	GO368317	25012	AT2G23420
	Target								
(23) ttgctggtcttggagttgcttg	miRNA	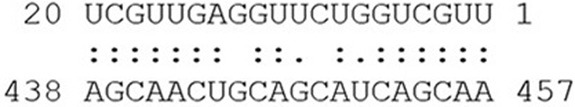	3	14.994	Translation	GQ03615_K16	BT115397	16822	AT4G00850
	Target								
(24) ttgttctgtagattttgaaac	miRNA	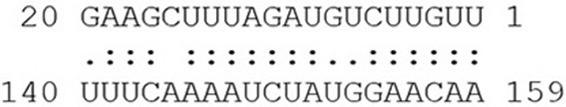	1	12.083	Cleavage	GQ03810_N15	EX425513	43571	AT2G18280
	Target								

*The header notation is identical with Table 1*.

An asterisk (^*^) indicates AdapTree transcriptome contig from Yeaman et al. (2016).

### Expression pattern of selected miRNA and genes explained by the environment

We chose the seed dormancy phenotype to investigate how environmental signals impinge on the expression of conserved miRNAs targeting genes related to seed dormancy and also that of key conservative genes conducive to the phenotype, thus collectively manipulating phenotypic variation. Gene phylogeny for *ARF10/16* showed that gymnosperm and model angiosperm species were separated into different clades except for *Picea*, which has a higher sequence similarity with angiosperms than species in the same taxonomic category (Figure S4). This indicates that *ARF10/16* is ancient and evolutionarily conserved within the plant kingdom. Conserved domain analysis showed that putative *ARF10* in *P. glauca* harbors an Aux_IAA super family domain (Figure S5). The pivotal activation function of ARF proteins is conferred by their four-domain architecture, including DNA binding region (a B3 and an ARF domain) and protein dimerization motifs (Ulmasov et al., 1999; Tiwari et al., 2003). Loss of the canonical four-domain structure has promoted functional shifts within the ARF family by disrupting either dimerization or DNA-binding capacities (Finet et al., 2013). As such, the putative *ARF10* in *P. glauca* may not correspond to its counterpart in *Arabidopsis* or the essential Aux_IAA domain is sufficient to have the function of *ARF10* in *P. glauca*. No homolog hits for *ABI3* and *DOG1* were obtained in some species (Table S2) and the phylogeny showed that they might have undergone substantial selection (Figure S4). The miR160 targets *ARF10* and its hairpin structure was computationally predicted (Figure S6).

The relative expression of aforementioned genes was displayed in Figure S7. As predicted, *AtDOG1, AtABI3, AtARF10*, and *AtARF16* were highly expressed in Cvi-0 than Col-0 (Figure S7, left panels). However, such pattern was not observed for gene counterparts in populations of *P. glauca* (Figure S7, first three panels on the right side), indicating that these genes in *P. glauca* may have different functions or that the studied phenotype in *P. glauca* is regulated by other unknown mechanisms. In the RDA triplot, the percentage of accumulated constrained eigenvalues showed that the first axis explained 43.8% variance (Figure S7, last panel), indicating that the major trends have been modeled by RDA. In addition to species and dev_phase, developmental temperature played an important role in the dispersion of developmental phases along the first axis and had high correlation with miR160 and ARF10/16 (Figure S7, last panel). As transcripts of *ARF10/16* are targeted by miR160 (Liu et al., 2013), the expression patterns of *ARF10/16* and miR160 were highly positively correlated with each other but negatively correlated with that of *DOG1* (Figure S7). In addition, the projection of the same developmental phases on the first axis was overlapped across populations in *P. glauca* (Figure S7). This suggests that the same phase between populations has more similarities in gene expression pattern than different phases within populations, and in turn, prompts the conservation of gene regulation at a temporal scale across populations.

## Discussion

To date, evolutionary analysis on miRNAs is almost comprehensive and profound throughout species in the tree of life (Nozawa et al., 2012), but there is a dearth of representative species in a subgroup of gymnosperms—conifers, from which flowering plants bifurcate, thus occupying an important taxonomic position. Although recently there have been sporadic reports on conifer small RNA sequences (e.g., Källman et al., 2013; Xia et al., 2015), no small RNA study is tailed to examine the reproductive period, during which small RNAs of the 24-nt size class are specifically and uniquely yielded (Nystedt et al., 2013) and the whole genome is highly methylated (Takuno et al., 2016). This implies a different landscape of small RNAs during conifer seed set. Furthermore, in multicellular organisms gene expression fine-tuned by miRNAs can decrease phenotypic variation (i.e., canalization) among individuals and even among cells, thus reducing conflicts among cells of different genetic background (Michod and Roze, 2001; Hornstein and Shomron, 2006; Flynt and Lai, 2008). Together, a supplemental characterization of sRNAs in conifer seed development therefore motivates and necessitates this study. In quest of molecular underpinnings of today's diversity of life and epigenetic phenomena involved in shaping responses to environmental signals, we rely on comparative studies of deeply conserved and known miRNA homologs as well as lineage- and population-specific sRNAs in two spermatophytes, *Picea glauca* and *A. thaliana*, to unravel, from evolutionary perspectives, the pattern of expression dynamics of sRNAs and miRNAs (also via comparing with deeply conserved ones in the plant kingdom), sRNA candidates targeting adaptive genes, and miRNA-gene regulation in one studied phenotype (i.e., seed dormancy). Understanding the underlying mechanism of local adaptation helps reveal evolutionary signatures that may comprise selective forces for adaptation and speciation. This study attests to the correlation between the sRNA (and miRNA) repertoire and the organismal complexity and provides a potential rationale to explore the evolutionary mechanism at the organism level via *de novo* methylation and sRNA interactions.

### Insights into miRNA evolution

Of 37 miRNA families that are deeply conserved in plant development throughout the plant kingdom (Willmann and Poethig, 2007), we identified 12 miRNA families at seed set between phyla (Table 1). Some are conserved across spermatophytes (i.e., miR163, −394, and −396), tracheophytes (i.e., miR159), and embryophytes (i.e., miR160, −166, −167, −171, −319, −390, and −408). The ubiquitously conserved miRNAs had significantly differential expression abundance between *P. glauca* and *Arabidopsis* as well as between populations within species (e.g., Figure 5IA vs. Figure 5IIA), and this observation is correlated to genome evolution (Hodgins et al., 2016). These mature miRNA sequences have a well-conserved consensus with minor variation at the 5′ or 3′ end between *P. glauca* and *Arabidopsis* (Table 1), and their targets may not always be conserved partially due to no homologs found in *P. glauca* (Table 1). This indicates that the cognate miRNA-target pairs may be acquired prior to the split of gymnosperms and angiosperms and have undergone natural selection. Conserved miRNAs in *Arabidopsis* (Table S8) were enriched and expressed across seed set (Figure 5IIA), while abundant sRNAs expressed throughout seed set in *P. glauca* did not belong to miRNA families documented on miRBase (Table S7). Nonetheless, their target genes were classified into the same GO categories (Figure 6). This indicates that spruce and Arabidopsis may use different sRNAs to attain similar regulations at molecular levels. Note that the known miRNA-gene pairs in Arabidopsis are associated with seed development and nodes in hormone-based GRNs (Figure 6C).

In general, ancient miRNAs are more highly and broadly expressed than younger ones (Fahlgren et al., 2007); while newly emerged miRNAs may be used as substrates for natural selection and form specific miRNA circuitry interplaying with GRNs, whereby adaptation and speciation occur (Chen and Rajewsky, 2007). During evolution, deleterious miRNA-target pairs are selected against and miRNAs by gradually decreasing the number of target genes are retained into GRNs (Chen and Rajewsky, 2007). Through constraining variance or mean gene expression, miRNAs render phenotypic traits evolvable as well as heritable (Wagner and Altenberg, 1996) and after selection, these miRNAs may improve fitness of phenotypes (Wu et al., 2009). However, most of young miRNAs appear to be selectively neutral [*S* = 0] (Fahlgren et al., 2010), because the majority of them does not have target genes or does not regulate targets in such a way that plant fitness can be enhanced, and they were quickly lost during evolution. In regulation of target genes with similar functions, different species may rely on variants of the same *MIRNA* families or produce distinct miRNAs through changes in miRNA precursor sequences and/or binding sites. From this study, the conserved miRNAs throughout the plant kingdom and lineage-specific sRNAs (Figures 4, 5) represent different levels of sRNA conservation serving for basic needs in plants and particular requirements in a given lineage, respectively. On the other hand, although members of plant *MIRNA* families are often highly similar, the same gene family varies in size and genomic structure between species, indicating dosage effects and spatiotemporal differences in *MIRNA* regulation (Li and Mao, 2007). There are miRNA orthologs that function quite differently and several miRNA isoforms have specific tissue expression patterns (Lelandais-Brière et al., 2009), indicative of functional divergence. We detected some miRNAs solely expressed in one population/ecotype, which were conserved in miRNA families on miRBase (Table 2). As per target gene function, target genes do not seem to be key components in GRNs and they primarily target repeats, intergenic sequences, and genes in cell-cell communication and signaling (Table 2). This suggests that altering mean miRNA expression in different plant populations is subject to fine-tuning to regulate the expression of target genes, especially for non-key ones in GRNs. The dosage balance hypothesis, a mechanism in maintaining *MIRNA* duplicates after WGDs (possibly also in small-scale tandem or segmental duplication events due to little impact on maintaining optimal stoichiometry among gene products; e.g., Birchler and Veitia, 2012), supports our inference, as key genes in GRNs are dosage sensitive, such that deletion of one copy may disrupt the whole network.

As requirements for a functional *MIRNA* are less demanding than a protein-encoding gene (PEG), *MIRNA* can easily evolve from various sources of unstructured transcripts, such as gene duplication, intergenic regions, transposable elements (Nozawa et al., 2012). As well, like PEGs, *MIRNA*s are subject to the same evolutionary processes, such as, substitution, insertion, recombination, and natural selection. There are three proposed models that explain the origination of *MIRNA*s, that is, transposable elements, inverted duplication of target genes, and random hairpin sequences with subsequent mutations, reviewed by Cui et al. (2016). A study in spruce supports the second model, evidenced by the precursor sequence of a copy of miR482 highly similar to nucleotide-binding site and leucine-rich repeat domains (*NBS-LRR*) genes (Xia et al., 2015). The third model emphasizes that a serendipitous DNA sequence transcribed and Dicer-processed yields a sRNA that interacts with a homolog target. Coevolution of miRNA sequence and target genes refines such interaction (Felippes et al., 2008). The miRNA-target complementarity in plants is extensive and stringent (Rhoades et al., 2002), suggestive of coevolution of miRNAs and their targets. Taken together, miRNA-target systems have undergone evolution in the context of genome evolution; despite divergences in miRNA sequences, the function of their target genes is largely of commonality (e.g., indirect evidence from Figure 6) and this supports similar molecular mechanisms and signaling cascades at seed set across phyla.

### Roles of sRNAs in local adaptation

To cope with the vagaries of environmental perturbations, plants have evolved mechanisms of stress avoidance (acclimation) and stress tolerance (adaptation) via well-honing gene expression. Reprogramming of gene expression via sRNAs is a major defense mechanism in plants as response to stresses (see a list of references in the Figure 7 legend). The sRNA pathways through (P)TGS intersect with mechanisms regulating different steps in the life of an mRNA, starting from transcription and ending at mRNA decay, in both nuclear and cytoplasmic compartments. Stresses usually trigger destabilization of chromatin states, manifested as changes in DNA methylation and reductions in nucleosome occupancy, and some of these processes involve miRNAs via the RdDM pathway and TE-associated 21-nt siRNAs (Dowen et al., 2012). Recently, a type of stress-induced, UTR-derived siRNAs in 24-nt long has been identified in *Brachypodium* and proposed to mask *cis*-elements (i.e., pre-mRNA) and direct the splicing machinery to use the correct splice site (Wang et al., 2015). Under stress, alternative splicing results in production of different splice variants and thus proteins with different functions required for survival, reviewed by Mastrangelo et al. (2012).

Studies in genomic basis of adaptation in long-lived organisms have largely focused on identifying intraspecific DNA variation and comparative gene expression that are associated with adaptation, reviewed by Prunier et al. (2016). In boreal and temperate regions, conifers with complex life cycles have been found to rely on the regulation of certain gene expression to defense against climatic stresses (Hornoy et al., 2015; Yeaman et al., 2016). These adaptive genes mainly participate in the process against abiotic stresses (e.g., cold injury, resistance to aridity, high salinity, UV-B, etc.) and fungal pathogens, growth cessation and bud set to synchronize with seasonal climatic changes. As such, divergence in gene expression possibly through stabilizing or directional selection (because studies in animals indicate that most gene expression variability may be deleterious and selected against [*S* = −∞]) is important to species adaptability and evolution. However, the long generation time (i.e., old age of sexual maturity) imposes limitations on natural selection and adaptive evolution for genes to cope with rapidly changing climate. Consequently, these organisms confront a real need to use epigenetics, owing to its merits of high flexibility, to deal with a wide range of environmental conditions throughout their life time. Our reported sRNAs (Figure 7A and Table 3) have opened a venue into better understanding evolutionary trajectories among small RNAs targeting adaptive genes (albeit not experimentally confirmed), as natural selection of sRNAs in different lineages and among populations within lineage may exhibit different evolutionary patterns and delving into these patterns for different organismal complexity helps uncover sRNA evolution in line with the complexity of organisms.

### Effects of the environment on phenotypic variation

The phenology or temporal control of the life cycle provides adaptive strategies to avoid adverse consequences in harsh environments at seed set and seedling establishment (Krämer, 2015). In the life cycle, temperature is of utmost importance in seed set, germination, and seedling stage due to epigenetic imprinting and their fragile state (Liu et al., 2016). At seed set, temperature signals are a critical selective pressure and have a strong influence on life history traits, such as timing of seed set and seed dormancy depth (Springthorpe and Penfield, 2015; Vidigal et al., 2016). Specifically, the temperature-mediated control of flowering has evolved to constrain the maternal environment for setting seeds to a specific temperature window, thus yielding seeds with dormancy variation. Maternal environments (e.g., temperature cues) have a persistent and transgenerational effect on the expression pattern of regulatory molecules in GRNs (e.g., *DOG1*, Chiang et al., 2013) and on the biogenesis of versatile miRNAs in life histories. The latter was confirmed by miRNAs (i.e., miR160) targeting key genes (i.e., *ABI3* and *ARF10/16*) that modulate timing of seed set and the dormancy state (Huo et al., 2016). In this study, the chosen populations of *P. glauca* and ecotypes of *Arabidopsis* are characterized by different fertilization timepoints, contrasting seed-set durations and ensuing phenotypic variation (i.e., dormancy intensities; Figure 1). We tested how the role of miRNA-gene interactions is at play for one study phenotype, seed dormancy, in which we chose a miRNA-gene pair (i.e., miR160-*ARF10/16*) and key genes (i.e., *ABI3* and *DOG1*) (Figure S7). Although the chosen gene may have different functions in *P. glauca*, our results reinforce the notion that environment factors, such as temperature have great impact on the expression of genotypes (miRNAs and genes).

## Conclusions and prospects

This study accentuated that roles of sRNAs in phenotypic variation are canalized at the reproductive period and coupled with species of different evolutionary time, which, metaphorically like writing on a palimpsest for the next generation, sets molecular imprinting on phenotypes that will be expressed in the adult stage for local adaptation. Through global analysis of small RNA dynamics across and among populations as well as within and between *P. glauca* and *Arabidopsis*, we demonstrated small RNA evolution and shed light upon its link to organismal complexity and genome evolution, as evidenced by (i) the expression pattern of deeply conserved miRNAs reflected different seed-set programs in *Arabidopsis* ecotypes (Figure 4); (ii) expression patterns of lineage-specific sRNAs enriched at the 21(or 22)-nt size class had no significant difference between populations but population-specific sRNAs were differently expressed in both *Arabidopsis* and *P. glauca* (Figure 5); (iii) sRNAs targeting reported adaptive genes were computationally predicted and both of them were not overlapped between the two species (Figure 7 and Table 3); and (iv) environmental factors (e.g., temperature) had significant influence on the expression of key genes and miRNAs at seed development in both species (Figure S7). Notwithstanding deeply conserved miRNA families play an important role across a plurality of plants, few studies isolate conserved miRNAs and sRNAs (including siRNAs and novel miRNAs) to comparatively uncover the frequent presence and absence of sRNAs in large quantities and its evolutionary significance shaped by natural selection.

In the future, we wish to advance the non-coding sRNA study in conifers with emphasis on the following two facets. One fascinating aspect is to examine the pattern of changes in DNA methylation in developing seeds and vegetative tissues among conifer populations and test whether the pattern in (de-)methylation is congruent with that observed in selection of sRNA sequences especially in the short size class (i.e., 21~22-nt). The motivation for this aspect comes from intriguing reports showing conspicuous 24-nt small RNAs (Nystedt et al., 2013) and a very high level of DNA methylation (Takuno et al., 2016) uniquely yielded at the reproductive period in conifers. Further on, comparative profiling of epigenetic changes in different populations has the potential to manifest how epigenetic mechanisms contribute to the gene expression regulation. The other aspect aims to investigate whether *MIRNA*s originate from TEs and undergo evolution coupled in time with genome evolution between species within spermatophyte and how the environment contributes to adaptive variation at molecular levels (e.g., epigenetic imprinting, genetic variants, etc.). This conception is emanated from our results concerning *MIRNA*s for abundant sRNAs in *Arabidopsis* (i.e., conserved miRNAs) containing more DNA repeat modules than those for enriched ones in *P. glauca* (Figure S8; also see Liu and El-Kassaby, 2017b). This may be linked to their giga-genome evolution, in the sense that genetic divergence is suppressed in conifers, thus leading to few WGDs. As our increased knowledge of macro-structural features of giga-genomes, this study will open new perspectives for understanding the evolutionary mechanism of sRNAs in association with *MIRNA*s, sRNA targets and genome evolution (e.g., avoiding pseudogenization via sub- or neofunctionalization, relative dosage or increased gene dosage) in forest trees.

## Data availability

The sRNA sequencing data has been deposited at Sequence Read Archive (SRA) in the National Center for Biotechnology Information (NCBI) under the accession numbers, SRP096198, SRP096194 and SRP072220.

## Author contributions

YL conceived this study, performed data analyses, and wrote the manuscript; YE coordinated the project.

### Conflict of interest statement

The authors declare that the research was conducted in the absence of any commercial or financial relationships that could be construed as a potential conflict of interest. The reviewer RN and handling Editor declared their shared affiliation.
