# Supplementary material for: Global Analysis of Small RNA Dynamics during Seed Development of Picea glauca and Arabidopsis thaliana Populations Reveals Insights on their Evolutionary Trajectories
Source: Front Plant Sci. 2017 Oct 4;8:1719. doi: 10.3389/fpls.2017.01719 (PMC5632664; doi:10.3389/fpls.2017.01719)
Supplement: Supplementary file 1 [file DataSheet1.pdf]

Frontiers in Plant Science

SUPPORTING INFORMATION

Global analysis of small RNA dynamics during seed development of *Picea glauca* and *Arabidopsis thaliana* populations reveals insights into their evolutionary trajectories

Yang Liu, Yousry A. El-Kassaby

Appendix 1: Additional Methods and Figures

Methods S1

Figures S1-S8

Methods S1

Exploratory data analysis

Exploratory data analysis (Martinez et al., 2011) was used to get a sense of the conserved and differentially expressed miRNAs in *P. glauca* and *Arabidopsis* and to delineate the results in bivariate plots with histograms along the diagnal (normality not required), which provided the frequency distribution of each variable, and scatterplot matrices using LOESS procedure in smoothing fitted curves was produced for each off-diagonal panel, which summarized the relationship between the variables (Carr et al., 1987).

Sequence module analysis

As repetitive DNA sequences (e.g., transposable elements, minisatellites, and palindromic sequences) are evolutionarily conserved and have significant functional importances, *MIRNA*s were analyzed by repeat DNA modules using ModuleOrganizer (Tempel and Talla, 2014). Based on our previous assumption regarding the correlation between seed set patterns and seed dormancy variation, we concentrated on miRNA-mRNA nodes and key conserved genes (i.e., *ARF10/16*, *ABI3*, and *DOG1*) responsible for seed dormancy modulation. To investigate those genes of interest annotated in other conifers or Arabidopsis but not yet documented in *P. glauca*, BLASTN analysis was performed using the *P. glauca* PlantGDB Putative Unique Transcripts (PUTs) database on ConGenIE (http://congenie.org/). These genes are detailed in Table S2. Multiple alignments of transcripts were conducted using ClustalW 2.0 (Larkin et al., 2007). To uncover mRNAs targeted by communally conserved miRNAs between phyla, tBLASTN was executed through Arabidopsis proteins with reference to conserved domains/motifs against translated (six frames) nucleotide database and a phylogram was constructed using the maximum likelihood algorithm with 1,000 rounds of bootstrapping, both at default settings, within the MEGA v6.0 software package (Tamura et al., 2013).

Additional Figures

Figure S1 Overview of conserved miRNAs expression patterns across seed-set phases of two ecotypes (Cvi-0 and Col-0) in four populations (Pop 1~4) of *P. glauca* (A) and two ecotypes of *Arabidopsis* (B)

Green lines are produced using linear regression; red solid and dashed lines are generated via non-parametric regression smoothing original data and the spread (i.e., using the square root of the variance function), respectively. An underscore line links population number to *P. glauca* seed-set phase (e.g., "Pop1_1" means Population 1 at seed-set timepoint 1) or ecotype to *Arabidopsis* seed-set phase (e.g., "Cvi_1" denotes Cvi-0 at timepoint 1).

Figure S2 Analyses of sRNA reads in *P. glauca*

Statistics of unique miRNA read numbers in stacked and compiled libraries by populations (A) and identification of the unique read number in populations (B).

Figure S3 Analyses of miRNA and sRNA reads in *Arabidopsis*

(A) Statistics of unique read numbers in stacked and compiled libraries for conserved miRNAs and sRNAs (conserved miRNA excluded). (B) Identification of the unique read number in conserved miRNAs and these sRNAs at early (Cvi_0~7 and Col_0~4) and late (Cvi_8~14 and Col_5~9) maturation.

Figure S4 Phylogenetic tree of homologs for four genes (*DOG1*, *ABI3*, *ARF10*, and *ARF16*) in gymnosperms and model angiosperms

As per tBLASTN, *DOG1* has a high sequence similarity with *TGA6/2* (i.e., basic leucine zipper TF involved in the activation of SA-responsive genes).

Figure S5 Nucleotide alignment of *ARF10* between *P. glauca* (BT119832) and *Cycas rumphii* (FN433183)

Figure S6 The hairpin structures of possible pgl-miR160s by a computational prediction

Figure S7 Relative expression of key genes in this study by time points at seed set and triplot diagram for RDA (last panel) between relative expression of miRNA-gene combinations (red lines) and environments (blue arrows)

dev_pattern denotes population or ecotype code and dev_phase is seed developmental phase.

Figure S8 The number of repeat modules per "conserved" *MIRNA* (A) and across *MIRNA*s (B) in *P. glauca* and *Arabidopsis*

One or three asterisks represent that the mean between two groups is significant ($P < 0.05$) or highly significant ($P < 0.001$), respectively, using Student *t*-test. The average length of analyzed mature sRNAs is 21.7 ± 1.36 nt and 21.6 ± 1.23 nt in *P. glauca* and Arabidopsis, respectively.

Figure S1

A) Bivariate plot profiles using conserved miRNAs expression across *Picea glauca* seed set phases of four populations

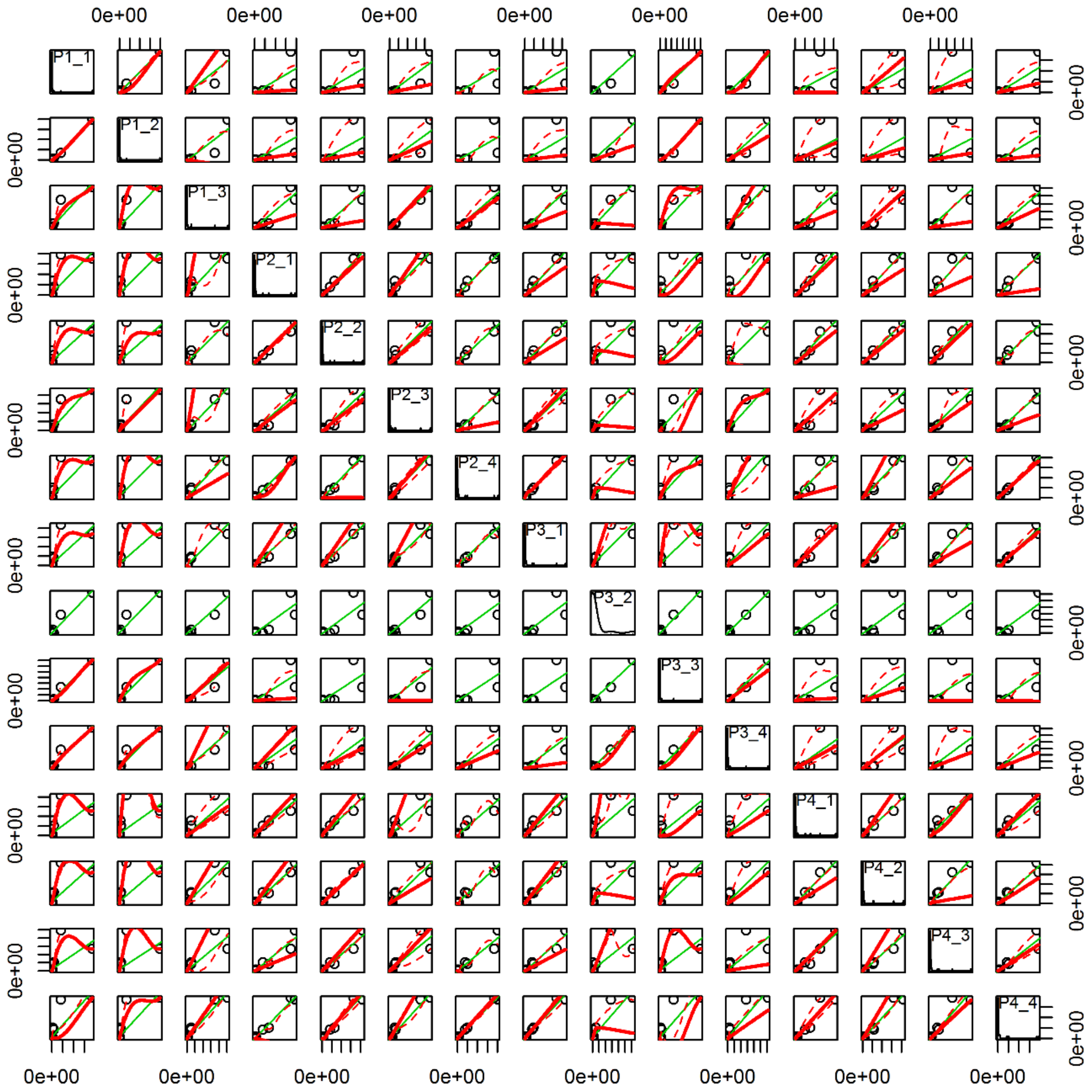

B) Bivariate plot profiles using conserved miRNAs expression across *Arabidopsis thaliana* seed-set phases of two ecotypes (Cvi-0 vs. Col-0), respectively

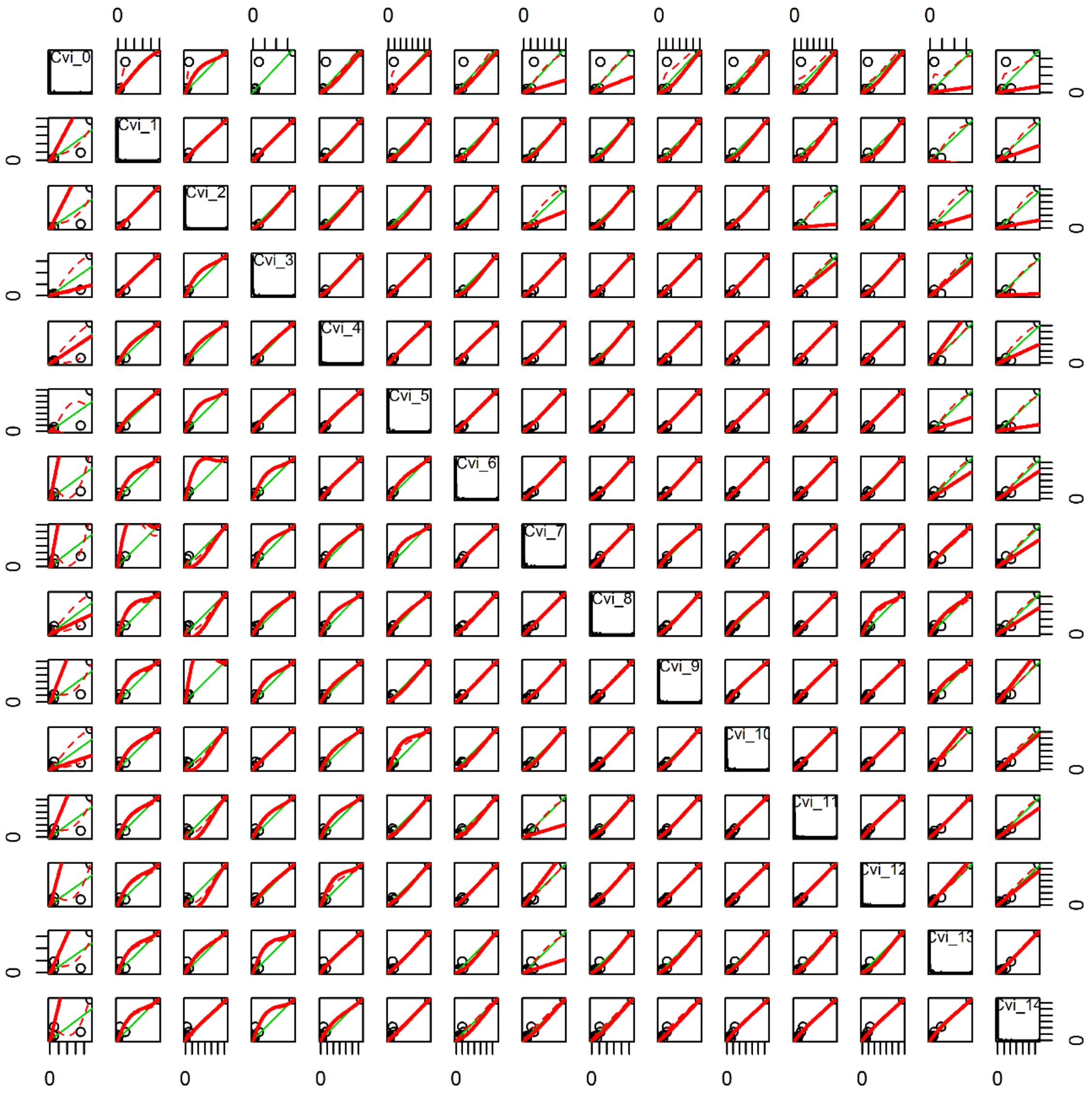

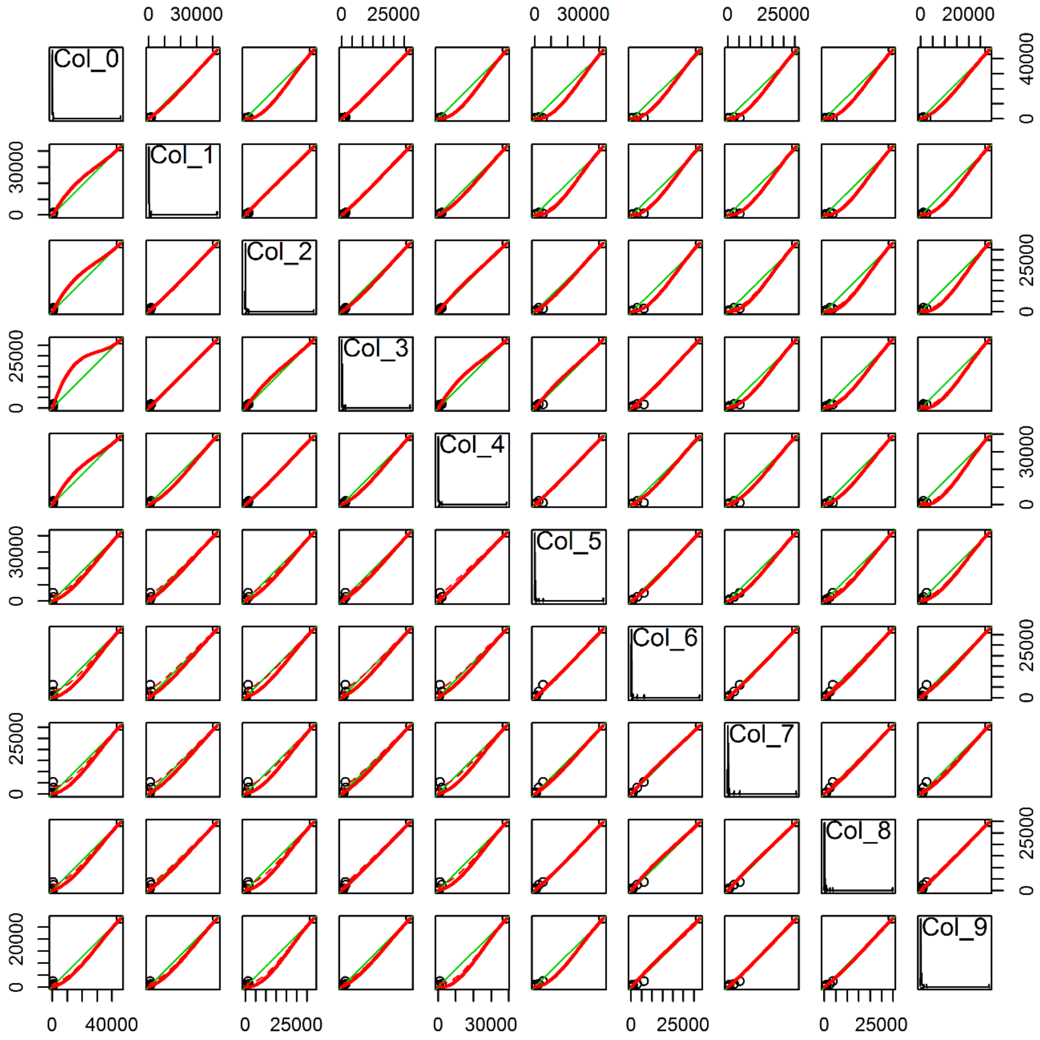
Col_0
Col_1
Col_2
Col_3
Col_4
Col_5
Col_6
Col_7
Col_8
Col_9
0 30000
0 25000
0 30000
0 25000
0 20000
0 40000
0 30000
0 25000
0 25000
0 30000
0 30000
0 25000
0 25000
0 25000
0 20000
0 40000
0 25000
0 30000
0 25000
0 25000

Figure S2

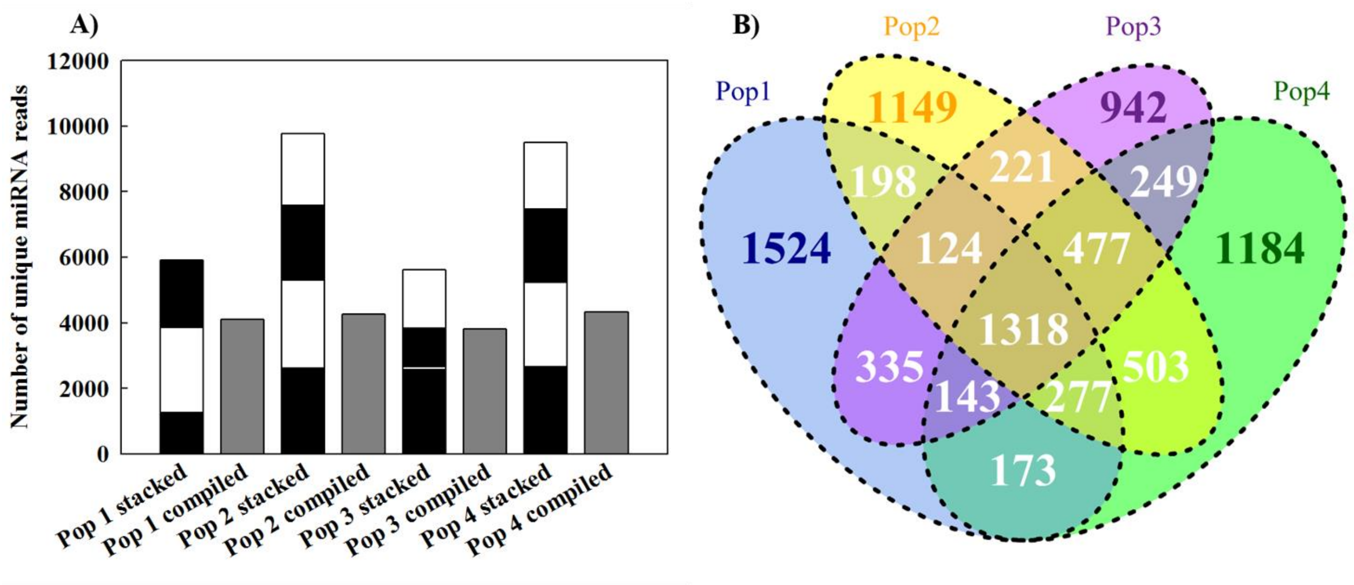
A)
Number of unique miRNA reads
12000
10000
8000
6000
4000
2000
0
Pop 1 stacked
Pop 1 compiled
Pop 2 stacked
Pop 2 compiled
Pop 3 stacked
Pop 3 compiled
Pop 4 stacked
Pop 4 compiled
B)
Pop1
Pop2
Pop3
Pop4
1149
942
1524
1184
198
221
249
124
477
1318
335
503
143
277
173

Figure S3

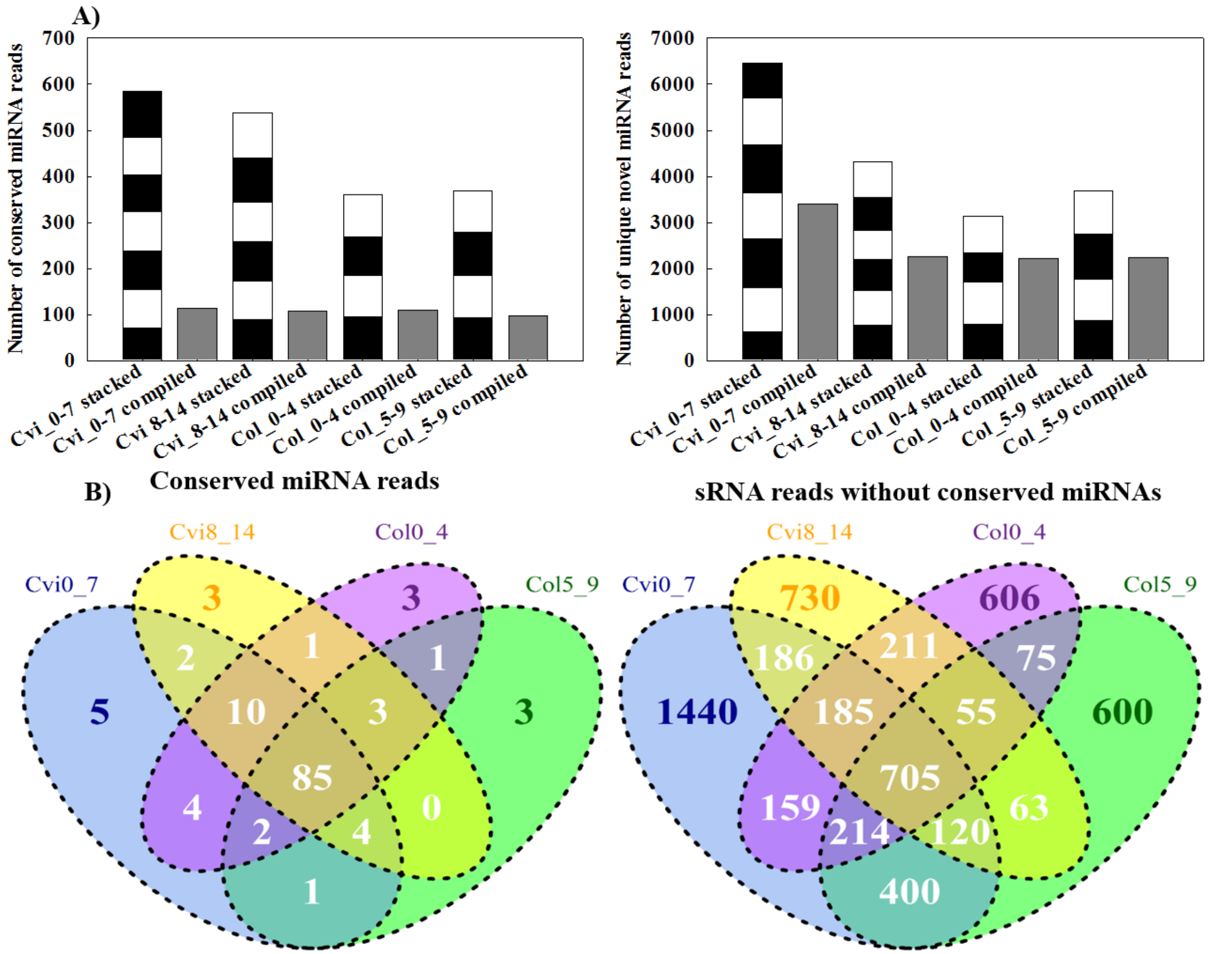
A)
Number of conserved miRNA reads
700
600
500
400
300
200
100
0
Cvi_0-7 stacked
Cvi_0-7 compiled
Cvi 8-14 stacked
Cvi_8-14 compiled
Col_0-4 stacked
Col_0-4 compiled
Col_5-9 stacked
Col_5-9 compiled
Number of unique novel miRNA reads
7000
6000
5000
4000
3000
2000
1000
0
Cvi_0-7 stacked
Cvi_0-7 compiled
Cvi_8-14 stacked
Cvi_8-14 compiled
Col_0-4 stacked
Col_0-4 compiled
Col_5-9 stacked
Col_5-9 compiled
B)
Conserved miRNA reads
Cvi0_7
Cvi8_14
Col0_4
Col5_9
5
3
3
3
2
1
1
10
3
85
4
0
2
4
1
sRNA reads without conserved miRNAs
Cvi0_7
Cvi8_14
Col0_4
Col5_9
1440
730
606
600
186
211
75
185
55
705
159
63
214
120
400

Figure S4

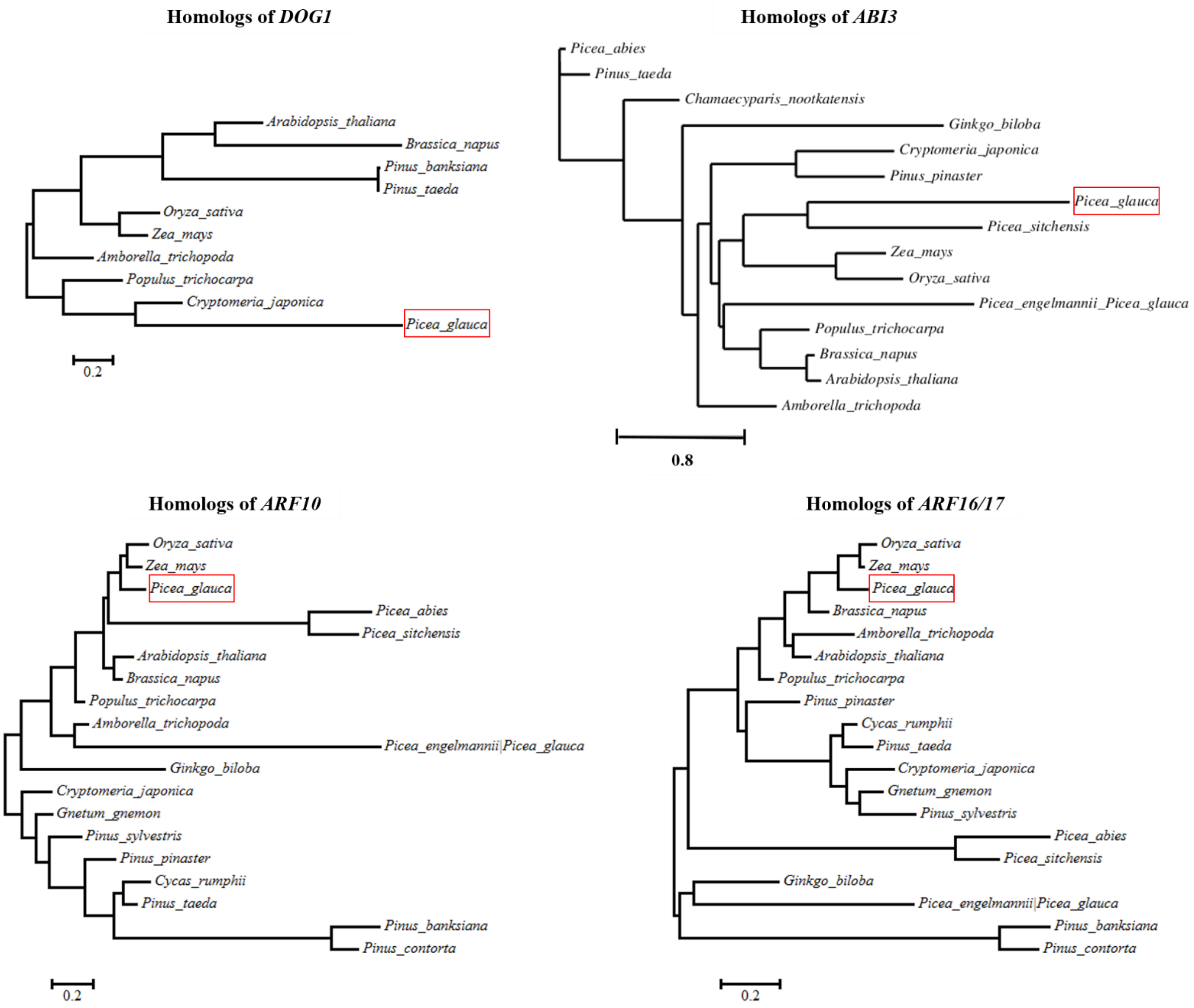
Homologs of DOG1
Arabidopsis_thaliana
Brassica_napus
Pinus_banksiana
Pinus_taeda
Oryza_sativa
Zea_mays
Amborella_trichopoda
Populus_trichocarpa
Cryptomeria_japonica
Picea_glauca
0.2
Homologs of ABI3
Picea_abies
Pinus_taeda
Chamaecyparis_nootkatensis
Ginkgo_biloba
Cryptomeria_japonica
Pinus_pinaster
Picea_glauca
Picea_sitchensis
Zea_mays
Oryza_sativa
Picea_engelmannii_Picea_glauca
Populus_trichocarpa
Brassica_napus
Arabidopsis_thaliana
Amborella_trichopoda
0.8
Homologs of ARF10
Oryza_sativa
Zea_mays
Picea_glauca
Picea_abies
Picea_sitchensis
Arabidopsis_thaliana
Brassica_napus
Populus_trichocarpa
Amborella_trichopoda
Picea_engelmannii|Picea_glauca
Ginkgo_biloba
Cryptomeria_japonica
Gnetum_gnemon
Pinus_sylvestris
Pinus_pinaster
Cycas_rumphii
Pinus_taeda
Pinus_banksiana
Pinus_contorta
0.2
Homologs of ARF16/17
Oryza_sativa
Zea_mays
Picea_glauca
Brassica_napus
Amborella_trichopoda
Arabidopsis_thaliana
Populus_trichocarpa
Pinus_pinaster
Cycas_rumphii
Pinus_taeda
Cryptomeria_japonica
Gnetum_gnemon
Pinus_sylvestris
Picea_abies
Picea_sitchensis
Ginkgo_biloba
Picea_engelmannii|Picea_glauca
Pinus_banksiana
Pinus_contorta
0.2

Figure S5

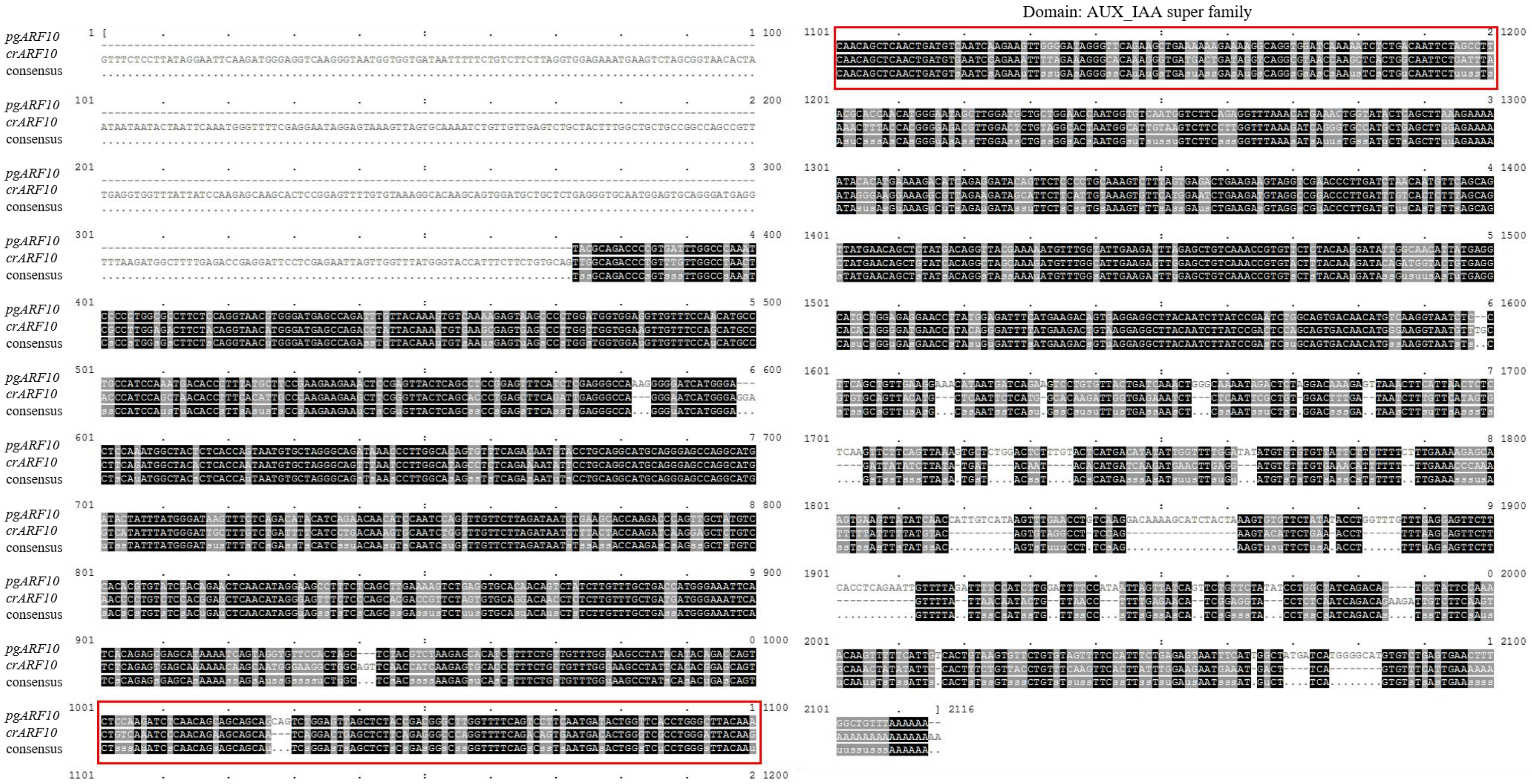

Figure S6

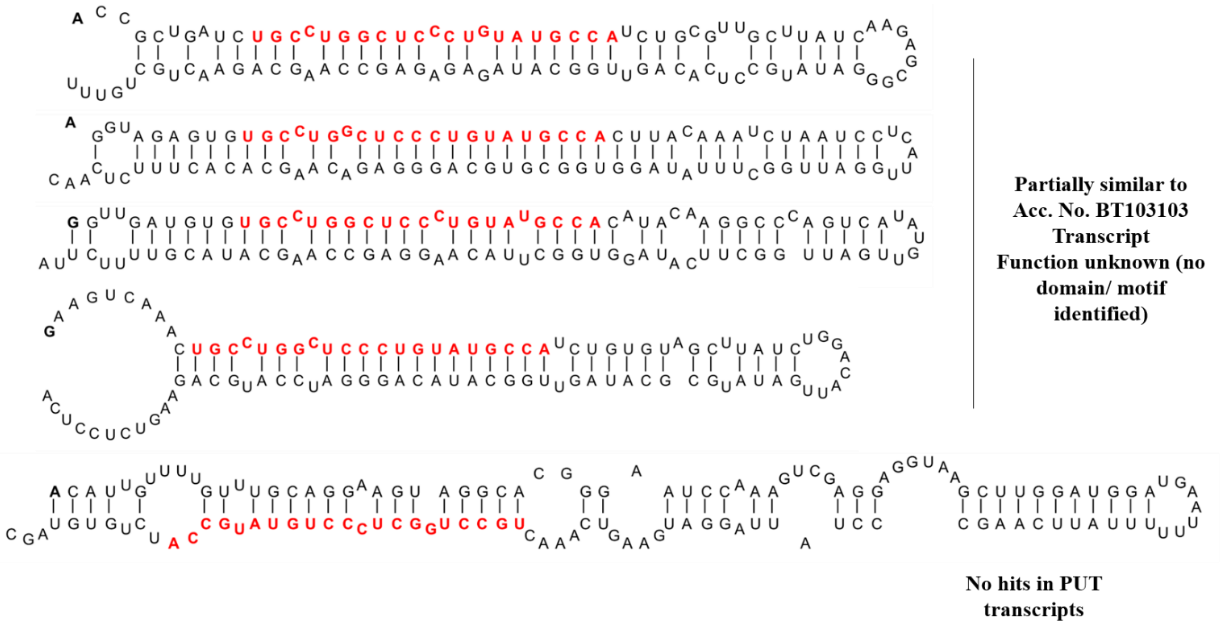
Partially similar to Acc. No. BT103103 Transcript Function unknown (no domain/ motif identified)
No hits in PUT transcripts

Figure S7

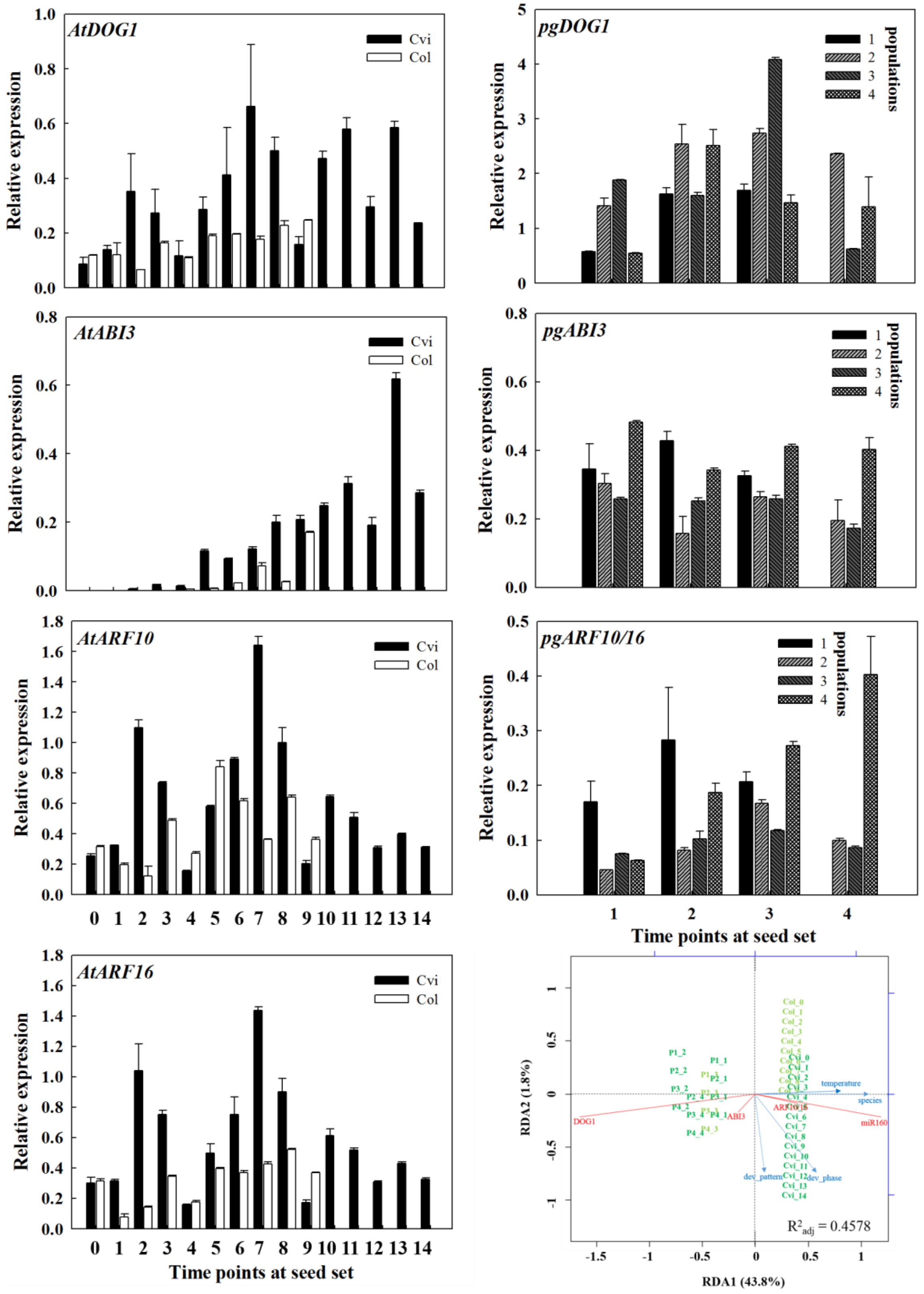
AtDOG1
Cvi
Col
Relative expression
pgDOG1
populations
1
2
3
4
Releative expression
AtABI3
pgABI3
AtARF10
pgARF10/16
AtARF16
Time points at seed set
RDA1 (43.8%)
RDA2 (1.8%)
DOG1
ABI3
miR160
temperature
species
dev_pattern
dev_phase
R^2_{adj} = 0.4578

Figure S8

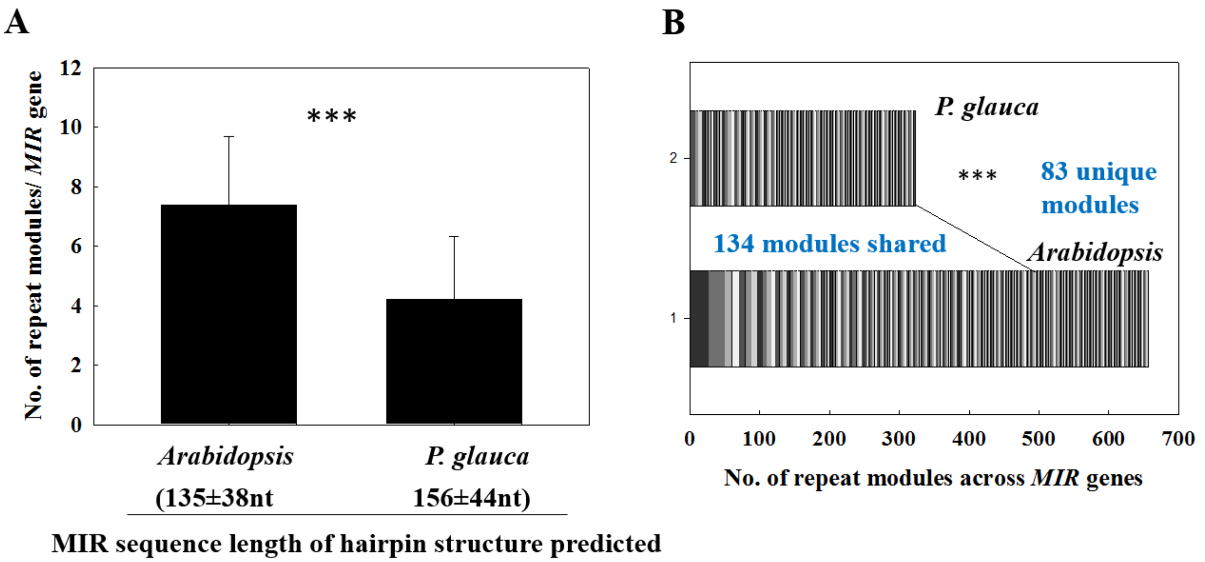

Description:

*MIRNA*s usually originate from non-protein-coding fragments. DNA repeat modules are evolutionarily conserved and using *MIRNA*s for enriched sRNAs throughout seed set in *P. glauca* (Table S7) and conserved miRNAs in *Arabidopsis* (Table S8), we found that there were significantly more repeat modules per *MIRNA* in *Arabidopsis* (7.4 ± 2.2) than in *P. glauca* (4.1 ± 2.1) (Figure S8A). 134 repeat modules were identical between *P. glauca* and *Arabidopsis*, while 83 repeats were unique in *Arabidopsis* (Figure S8B). The total number of repeat modules in *Arabidopsis* (656) was significantly higher than in *P. glauca* (323) (Figure S8B). These differences indicate that their *MIRNA*s are subject to divergent evolution, in the sense that transposons, short repeats and other insertions seem targets of differential demethylation (Gehring et al., 2009), allowing changes in the expression pattern of imprinted genes (Pignatta et al., 2014). Two possibilities may result in the *MIRNA* evolution: pre-existing *MIRNA*s evolve and update their functions or new *MIRNA*s emerge to acquire new functions.

Additional References

Carr, D.B., Littlefield, R.J., Nicholson, W.L., and Littlefield, J.S. (1987). Scatterplot matrix techniques for large N. *Journal of the American Statistical Association* 82**,** 424-436.

Gehring, M., Bubb, K.L., and Henikoff, S. (2009). Extensive demethylation of repetitive elements during seed development underlies gene imprinting. *Science* 324**,** 1447-1451.

Larkin, M.A., Blackshields, G., Brown, N.P., Chenna, R., Mcgettigan, P.A., Mcwilliam, H., Valentin, F., Wallace, I.M., Wilm, A., Lopez, R., Thompson, J.D., Gibson, T.J., and Higgins, D.G. (2007). Clustal W and Clustal X version 2.0. *Bioinformatics* 23**,** 2947-2948.

Martinez, W.L., Martinez, A.R., and Solka, J.L. (2011). *Exploratory data analysis with MATLAB (2nd ed.).* Boca Raton, Florida: CRC Press.

Pignatta, D., Erdmann, R.M., Scheer, E., Picard, C.L., Bell, G.W., and Gehring, M. (2014). Natural epigenetic polymorphisms lead to intraspecific variation in *Arabidopsis* gene imprinting. *eLIFE* 3**,** e03198.

Tamura, K., Stecher, G., Peterson, D., Filipski, A., and Kumar, S. (2013). MEGA6: Molecular evolutionary genetics analysis version 6.0. *Molecular Biology and Evolution* 30**,** 2725-2729.

Tempel, S., and Talla, E. (2014). Visual ModuleOrganizer: A graphical interface for the detection and comparative analysis of repeat DNA modules. *Mobile DNA* 5**,** 9.
